# Variation in acute myocardial infarction management by kidney function across hospitals in England: a cross-sectional study using the Myocardial Ischaemia National Audit Project (MINAP)

**DOI:** 10.1136/bmjopen-2024-096991

**Published:** 2025-05-16

**Authors:** Patrick Bidulka, Clive Weston, Mark de Belder, John Deanfield, Rob Konstant-Hambling, Richard Grieve, David Adlam, Dorothea Nitsch

**Affiliations:** 1 Non-Communicable Disease Epidemiology, London School of Hygiene and Tropical Medicine Faculty of Epidemiology and Population Health, London, UK; 2 Glangwili General Hospital, Carmarthen, UK; 3 National Institute for Cardiovascular Outcomes Research (NICOR), NHS Arden & Greater East Midlands Commissioning Support Unit, Leicester, UK; 4 Institute of Cardiovascular Sciences, University College London, London, UK; 5 NHS England, Redditch, UK; 6 Department of Health Services Research and Policy, London School of Hygiene and Tropical Medicine, London, UK; 7 Department of Cardiovascular Sciences and NIHR Biomedical Research Centre, University of Leicester, Leicester, UK

**Keywords:** Audit, Health Services, Ischaemic heart disease

## Abstract

**Abstract:**

**Objectives:**

We hypothesised that there is substantial variation in acute myocardial infarction (AMI) treatment across English hospitals, particularly for people hospitalised for non-ST-elevation myocardial infarction (NSTEMI) and with reduced kidney function. This study aimed to describe this variation at the hospital and the individual level to understand treatment variation and potential disparities in AMI management among people with reduced kidney function.

**Design:**

Cross-sectional study.

**Setting:**

Secondary care in England.

**Participants:**

People hospitalised for AMI (ST-elevation myocardial infarction (STEMI) or NSTEMI) in English hospitals and captured in the Myocardial Ischaemia National Audit Project, 2014 to 2019. Kidney function was defined using estimated glomerular filtration rate (eGFR) derived from the serum creatinine recorded within 24 hours of AMI admission.

**Outcome measure:**

The primary outcome was recorded invasive cardiac intervention (at least one of angiography, percutaneous coronary intervention and coronary artery bypass graft) compared with conservative management.

**Results:**

We included 361 259 people with a first hospitalisation for AMI (STEMI or NSTEMI) at 209 hospitals for hospital-level analyses and 292 572 people with complete covariable data at 207 hospitals for individual-level analyses. We found substantial variation in the mean proportion of people with NSTEMI managed invasively across hospitals in England. At the individual level, using multivariable logistic regression to derive adjusted predicted probabilities to describe the association between kidney function and AMI management (invasive vs conservative management), we found that people had a lower adjusted predicted probability of being treated with invasive cardiac management with worsening eGFR range, particularly for NSTEMI cases (eGFR range 2: 76.6% (95% CI 76.3 to 76.8) vs eGFR range 5: 44.5% (95% CI 41.2 to 47.5)).

**Conclusions:**

There is substantial AMI treatment variation across hospitals in England, particularly among people hospitalised for NSTEMI with reduced kidney function. Further research is needed to evaluate the comparative effectiveness of NSTEMI management strategies for complex patients.

**STRENGTHS AND LIMITATIONS OF THIS STUDY**

- We used Myocardial Ischaemia National Audit Project data which is a national audit dataset describing the acute myocardial infarction (AMI) management pathway for most AMI hospitalisations in England and Wales.
- We described AMI management variation at both the hospital and the individual levels, while most previously published studies have focused on the individual level.
- We defined kidney function using admission serum creatinine measured in secondary care rather than serum creatinine recorded in primary care, resulting in potential misclassification of baseline kidney function; however, serum creatinine at admission is what cardiologists use when considering AMI management strategies.
- We did not include other secondary care data sources (eg, Hospital Episode Statistics), meaning we may be missing some AMI hospitalisations during the study time period in England.

## Introduction

 People with chronic kidney disease (CKD) are at substantially increased risk of acute myocardial infarction (AMI)^1 2^ and worse outcomes after AMI compared with the general population.^3^ Randomised controlled trials (RCTs) which have driven improvements in AMI treatment and outcomes^4^,^6^ frequently exclude people with kidney impairment,^7^ making it challenging to generalise findings from these trials to this high-risk population.

National and international guidelines recommend early invasive cardiac management strategies, namely, angiography and percutaneous coronary intervention (PCI) if indicated, for almost all ST-elevation myocardial infarction (STEMI) cases and for non-ST-elevation myocardial infarction (NSTEMI) cases judged to be at high risk of mortality.^8^,^10^ Specifically, the National Institute for Health and Care Excellence (NICE) recommends balancing benefits versus risks of invasive cardiac management, particularly for those at elevated risk of complications (eg, bleeding and acute kidney injury (AKI)) due to comorbidities such as CKD.^8^ Serum creatinine is a key element of contemporary risk calculators used to inform the decision for invasive versus conservative cardiac management.^11^

Observational studies and subgroup analyses from RCTs have suggested that people with reduced kidney function may benefit from invasive cardiac management despite the risks.^312^,^17^ However, there is a lack of RCTs comparing NSTEMI management strategies specifically in this population. The lack of decisive evidence favouring either invasive or conservative cardiac management strategies among people with reduced kidney function may partly explain the well-described ‘individual-level’ association between worsening kidney function and decreased odds of receiving invasive cardiac management for NSTEMI and, to some extent, STEMI in England.^14^,^16^ This lack of evidence may also explain, in part, the overall variation in invasive versus conservative cardiac management strategies, particularly for NSTEMI, observed across hospitals serving the general population in England.^18 19^

This study aimed to describe variation in AMI management for people with reduced kidney function in England. Specifically, our objectives were as follows:

1. Describe the population hospitalised for AMI and the crude proportion managed with invasive versus conservative cardiac management (stratified by AMI subtype and kidney function) aggregated at the hospital level.
2. Repeat the objective 1 descriptive analysis at the individual level.
3. Describe the association between reduced kidney function and AMI management strategy for STEMI and NSTEMI cases separately.

## Materials and methods

### Study design and data source

We used a cross-sectional study design to investigate AMI management variation at the hospital and individual levels according to kidney function, using data from the Myocardial Ischaemia National Audit Project (MINAP)—a prospective national audit programme.^20^ MINAP collects detailed information on patient demographics, admission timings and methods, in-patient care including the timeliness of invasive coronary procedures, previous and new drug prescriptions, comorbidity data and discharge or in-hospital death data.^18 20^ We considered the entire AMI hospitalisation as a snapshot of care received for each person included in this cross-sectional study.

### Study population

We included hospitals in England reporting at least one person hospitalised for AMI (STEMI or NSTEMI) in MINAP between 1 January 2014 and 31 March 2019. We excluded hospitalisations across all hospitals for unstable angina or any other diagnosis (threatened myocardial infarction, chest pain uncertain cause, myocardial infarction unconfirmed). We included only one hospitalisation per individual (the earliest in the study time period), in both the aggregated and individual-level analyses. Individual-level analyses additionally excluded people with missing covariable data for complete case analyses.

### Exposure of interest

Our primary exposure of interest was reduced kidney function, which we defined using the estimated glomerular filtration rate (eGFR). We derived the eGFR using the serum creatinine measured within 24 hours of AMI hospitalisation recorded in MINAP. Serum creatinine was converted to eGFR using the CKD Epidemiology Collaboration (CKD-EPI) formula,^21^ without adjustment for ethnicity. Since we do not have other important clinical measures like elevated albumin-to-creatinine ratio and we are not applying the chronicity criteria of having two measures <60 mL/min/1.73 m^2^ separated by 3 months, we cannot determine CKD stage. However, we use the same Kidney Disease Improving Global Outcomes guidelines for CKD cut points^22^ to define eGFR ranges in this study. These were eGFR range 1 (≥90 mL/min/1.73 m^2^), range 2 (60–89), range 3a (45–59), range 3b (30–44), range 4 (15–29) and range 5 (0–14). Reduced kidney function was defined as having eGFR ranges 3a–5 or coded chronic renal failure. We categorised people with missing eGFR separately but assumed that they did not have reduced kidney function in analyses which stratified by kidney function (eGFR ranges 1–2, ie, no reduced kidney function, vs eGFR ranges 3–5/coded renal failure, ie, reduced kidney function).^23^ If a person was transferred between hospitals during an AMI hospitalisation, we used the serum creatinine recorded at the first hospital to which the person was admitted to define eGFR range, since subsequent serum creatinine measures are likely to be biased upward from baseline kidney function due to AKI either co-incident or resultant from the AMI and its management.

### Outcomes

Our main outcome of interest was invasive cardiac management, defined as angiography and, if indicated, primary PCI or coronary artery bypass graft during the index AMI hospitalisation. Those not recorded as receiving invasive cardiac management were considered treated with conservative management. Several MINAP variables were used to define this outcome (online supplemental table 1).

### Covariables

We selected several variables which we expect to confound the association between reduced kidney function and AMI management strategy. These variables were sex, age, ethnicity (White, Asian, Black, Mixed/other), comorbidities (previous AMI, angina, hypertension, hypercholesterolaemia, peripheral vascular disease, cerebrovascular disease, chronic obstructive pulmonary disease, heart failure, type 2 diabetes mellitus), co-prescriptions (renin-angiotensin system inhibitors, beta-blockers, statins), smoking status and year of AMI hospitalisation. We compared these covariables across hospitals and adjusted for them in multivariable models. While MINAP reports data in financial years (April to March), we reported year of AMI hospitalisation in calendar years (January to December), meaning that the final year of data collection only included AMI hospitalisations recorded between January and March.

We stratified analyses by AMI subtype (STEMI, NSTEMI), defined using a previously developed algorithm for MINAP data (online supplemental table 2). The main analyses were also restricted to hospitals which offer PCI all the time. The MINAP data distinguished between individual hospitals, rather than groups of hospitals at the NHS trust level (online supplemental table 3). Secondary analyses considered all hospitals submitting data, including those which offer PCI (1) sometimes and (2) in exceptional circumstances or never (online supplemental table 3).

### Analysis

#### Objective 1 – descriptive analysis at the hospital level

We described hospital-level variation in aggregated individual-level covariables using the median proportions of people with each covariable across hospitals (reported as median, interquartile range (IQR)). Aggregate hospital-level population descriptors were presented overall and by PCI availability at the included hospitals. People who were transferred between hospitals during the same AMI event were allocated to the first hospital to which they were admitted.

For hospitals offering PCI all the time, we then described the proportion of people in each hospital who were managed invasively versus conservatively using stacked bar charts. We stratified this descriptive analysis by kidney function (no evidence of reduced kidney function (eGFR≥60 mL/min/1.73 m^2^ or eGFR missing) vs evidence of reduced kidney function (eGFR<60 mL/min/1.73 m^2^)).

In secondary descriptive analyses, we described hospital variation in invasive versus conservative cardiac management by plotting the proportion of individuals with reduced kidney function (x-axis) versus (1) the proportion of STEMI cases reported as being managed with an invasive cardiac strategy and (2) the proportion of NSTEMI cases reported as being managed with an invasive cardiac strategy (y-axis), with each point representing a hospital. We distinguished hospitals in these plots by PCI availability to understand the impact of interventional service availability on hospital-level variation. To better understand how clinically important comorbidities may impact variation in invasive cardiac intervention among the subpopulation where clinical uncertainty is greatest (NSTEMI cases with reduced kidney function),^10^ we also plotted hospital-level variation in invasive cardiac treatment restricted to this population including the following as the independent variable (x-axis): (1) % of cases aged ≥80 years, (2) % of cases with previous myocardial infarction, (3) % of cases with prevalent diabetes mellitus and (4) % of cases with prevalent heart failure.

#### Objective 2 – descriptive analyses at the individual level

At the individual level, we described the overall study population hospitalised for AMI at any hospital in England. We compared those with complete versus incomplete covariable and serum creatinine (eGFR) information to inform our analysis approach.

We then described those who were hospitalised for AMI at a hospital offering PCI services all the time, stratified by eGFR range. This analysis included complete cases only. We also described the population with coded chronic renal failure according to their eGFR at admission to better understand the characteristics of this population.

#### Objective 3 – the association between kidney function and acute myocardial infarction management strategy

We focused the analyses for objective 3 on the study population who were hospitalised at a hospital offering PCI services all the time with no missing covariable data. We used a mixed effects multivariable model to distinguish between AMI management variation due to individual-level factors versus variation at the hospital level using a mixed effects multivariable model built in three stages. For both STEMI and NSTEMI cases, we modelled the outcome (invasive vs conservative cardiac management) including (1) hospital as a random effect (to describe AMI management variation explainable by differences between hospitals), (2) model 1 plus eGFR range as a fixed effect (to understand how individual’s eGFR range accounts for some of the AMI management variation across hospitals) and (3) model 2 plus all other potential confounders as fixed effects (to understand how eGFR range and all other potential confounders account for the AMI management variation across hospitals). The *intracluster coefficient*, which is the ratio of the between-cluster variability and the sum of the within-cluster and between-cluster variabilities^24^ (ie, the proportion of the variation in the outcome explained by the hospital-level variation), was used to quantify the variation due to between hospital differences across these three different models. We calculated the adjusted predicted percentages of receiving invasive versus conservative cardiac management by eGFR range from model 3 to understand individual-level variation in AMI treatment, since percentages are easier to interpret than ORs.^25 26^

### Patient and public involvement

Patients were not involved in the design, analysis or write-up of this particular study. However, patients were involved in previous work^16 27^ which informed this study design and analysis, as well as the design of the overall funded project (see funding details).

## Results

### Objective 1 – descriptive analysis at the hospital level

**Figure 1:**
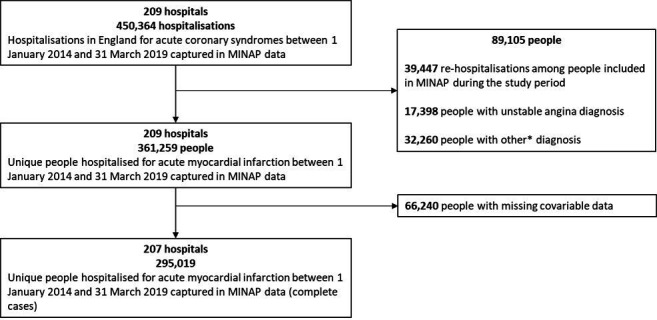
Flow diagram showing study population selection. *Other diagnoses include threatened myocardial infarction, chest pain (uncertain cause), myocardial infarction unconfirmed, other diagnosis (not myocardial infarction). MINAP, Myocardial Ischaemia National Audit Project.

Of the 450 364 hospitalisations for acute coronary syndromes at 209 hospitals in England between 1 January 2014 and 31 March 2019, we included 361 259 people with a first hospitalisation for AMI (STEMI or NSTEMI) at 209 hospitals for hospital-level analyses and 292 572 people with complete covariable data at 207 hospitals for individual-level analyses (figure 1). Of the 361 259 people included in the hospital-level analyses, 26 351 (7%) were transferred to at least one other hospital within the same AMI event (online supplemental table 4).

Of the 209 hospitals included in the dataset, 120 did not offer PCI services, 38 offered PCI sometimes and 51 offered PCI all the time. Aggregated at the hospital level, a higher median proportion of people were female who first presented to hospitals where PCI was only available in exceptional circumstances or not at all (37%, IQR 34 to 39) compared with hospitals with PCI always available (29%, IQR 26 to 30) (table 1). The median proportion of people aged 80–89 and 90+ years was higher among hospitals with PCI not available (25%, IQR 22 to 26 and 8%, IQR 6 to 11, respectively) compared with hospitals with PCI always available (16%, IQR 14 to 20 and 3%, IQR 2 to 6, respectively). The median proportion of people with comorbidities also tended to be higher among hospitals with PCI not available compared with hospitals with PCI always available (eg, angina (26%, IQR 19 to 32 (PCI not available) vs 13%, IQR 9 to 20 (PCI always available)) and previous myocardial infarction (23%, IQR 21 to 26 (PCI not available) vs 15%, IQR 14 to 18 (PCI always available))).

**Table 1:** Aggregate population characteristics at the hospital level for people hospitalised for AMI between 2014 and 2019 in England

	All hospitals	PCI not available	PCI available sometimes	PCI available all the time
	N=**209**	N=**120**	N=**38**	N=**51**
Total number of people admitted for STEMI or NSTEMI (2014–19), n=	295 019	95 503	54 345	145 171
Number of people admitted per year, median number of people (IQR)				
2014	271 (129–436)	175 (102–280)	317 (188–432)	601 (437–841)
2015	282 (137–443)	167 (99–282)	306 (222–424)	603 (460–853)
2016	253 (131–414)	168 (93–258)	305 (192–386)	595 (480–873)
2017	258 (147–454)	173 (77–252)	306 (208–408)	643 (526–882)
2018	244 (153–460)	174 (103–237)	316 (254–410)	641 (501–911)
2019 (January to March only)	63 (33–108)	42 (17–63)	82 (63–100)	161 (114–221)
Female	35 (30–38)	37 (34–39)	35 (32–37)	29 (26–30)
Age (years)				
50–59	16 (13–19)	14 (13–16)	16 (14–18)	21 (19–23)
60–69	22 (20–24)	21 (19–23)	21 (19–23)	25 (23–26)
70–79	25 (23–27)	25 (23–27)	25 (24–27)	24 (22–25)
80–89	23 (19–26)	25 (22–26)	23 (21–26)	16 (14–20)
90+	7 (4–9)	8 (6–11)	8 (5–10)	3 (2–6)
Missing	0 (0–0)	0 (0–0)	0 (0–0)	0 (0–0)
Ethnicity				
White	86 (64–96)	88 (64–96)	86 (64–94)	85 (63–95)
Black, Asian, mixed or other	3 (1–11)	2 (1–7)	5 (1–13)	4 (1–11)
Missing	4 (1–15)	5 (0–16)	2 (1–15)	4 (1–13)
eGFR range at AMI hospitalisation				
1–2	67 (62–72)	65 (62–69)	66 (61–72)	74 (71–78)
3a–3b	23 (19–26)	25 (22–27)	24 (21–27)	17 (16–20)
4–5	6 (4–7)	6 (5–8)	7 (5–8)	4 (3–5)
Missing	2 (1–4)	2 (1–4)	2 (1–3)	2 (1–5)
Comorbidities				
Angina	23 (16–29)	26 (19–32)	23 (18–29)	13 (9–20)
Cerebrovascular disease	9 (6–11)	10 (7–12)	8 (7–10)	6 (3–8)
COPD	16 (13–19)	18 (15–21)	16 (15–19)	13 (11–16)
Type 2 diabetes mellitus	25 (23–27)	26 (24–27)	27 (24–29)	20 (19–23)
Heart failure	6 (4–9)	7 (6–10)	7 (6–8)	3 (2–6)
Hypercholesterolaemia	30 (21–40)	30 (21–42)	31 (25–36)	29 (22–40)
Hypertension	52 (47–57)	54 (49–60)	52 (48–56)	48 (44–52)
Myocardial infarction	22 (17–25)	23 (21–26)	22 (20–26)	15 (14–18)
Peripheral vascular disease	4 (3–6)	5 (3–6)	4 (3–5)	3 (2–5)
Renal failure	8 (4–10)	8 (5–11)	9 (7–10)	4 (2–7)
Previous coronary interventions				
PCI	11 (9–14)	11 (9–15)	12 (10–16)	11 (9–13)
CABG	7 (6–9)	8 (6–9)	8 (7–9)	5 (4–6)
Prescriptions pre-AMI hospitalisation				
Beta-blocker	28 (23–32)	30 (27–34)	30 (28–33)	20 (15–23)
RASi	37 (30–41)	38 (35–42)	39 (35–43)	28 (21–32)
Statin	42 (35–48)	44 (40–49)	44 (39–48)	32 (27–36)
Smoking status				
Non-smoker	37 (34–43)	37 (34–44)	38 (35–45)	37 (32–40)
Ex-smoker	33 (28–37)	34 (31–37)	33 (27–37)	29 (26–32)
Current smoker	21 (18–27)	20 (17–24)	20 (16–24)	29 (24–33)
Missing	4 (1–9)	5 (2–10)	4 (1–9)	4 (1–9)

Reported as the median proportion, %, (IQR) across hospitals of people with each characteristic, unless otherwise specified. Presented across all hospitals and stratifying hospitals according to PCI availability.

eGFR ranges (mL/min/1.73 m2): range 1 (≥90), range 2 (60–89), range 3a (45–59), range 3b (30–44), range 4 (15–29) and range 5 (0–14).

AMI, acute myocardial infarction; CABG, coronary artery bypass graft; COPD, chronic obstructive pulmonary disease; eGFR, estimated glomerular filtration rate; NSTEMI, non-ST-elevation myocardial infarction; PCI, percutaneous coronary intervention; RASi, renin-angiotensin system inhibitor; STEMI, ST-elevation myocardial infarction.

**Figure 2:**
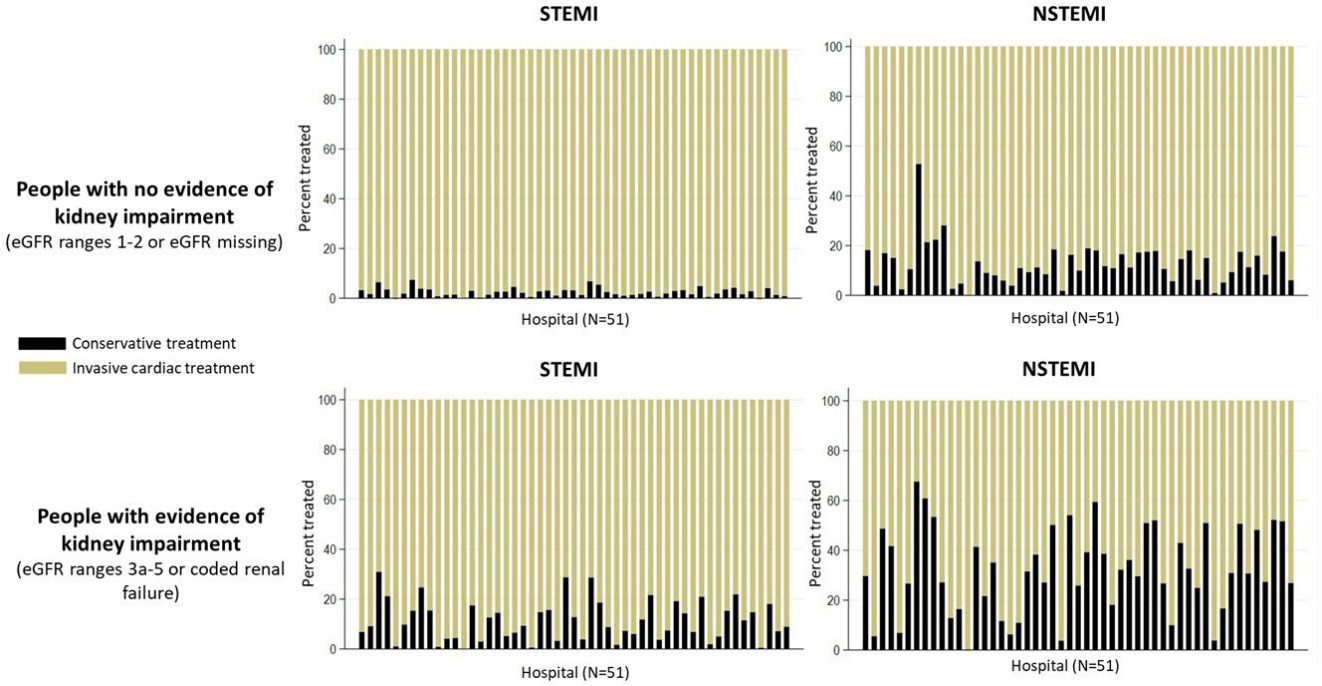
Hospital variation in invasive cardiac treatment versus conservative treatment among centres with percutaneous coronary intervention (PCI) always available (n=51), stratified by acute myocardial infarction (AMI) subtype (ST-elevation myocardial infarction (STEMI) and non-ST-elevation myocardial infarction (NSTEMI)) and level of kidney function (no evidence of reduced kidney function and evidence of reduced kidney function). eGFR, estimated glomerular filtration rate.

In hospitals with PCI always available (n=51), we observed substantial variation in the percent reported as treated with invasive versus conservative cardiac management, particularly for people hospitalised for NSTEMI with reduced kidney function (figure 2). There was very little variation in reported AMI management for people hospitalised for STEMI with no evidence of reduced kidney function.

We observed substantial variation across all hospitals in England (with varying PCI availability) in the proportion of people reported as receiving invasive versus conservative cardiac management for both STEMI and NSTEMI (online supplemental figure 1). For both STEMI and NSTEMI, there was a negative association between the proportion of AMI cases reported as receiving invasive cardiac management and the proportion of AMI cases with an admission eGFR<60 mL/min/1.73 m^2^. However, the variation in reported invasive cardiac management between hospitals for STEMI hospitalisations was dependent on PCI availability: an average (SD) proportion of 0.58 (0.19) and 0.77 (0.16) with STEMI were reported as receiving an invasive cardiac management strategy at hospitals with PCI available in exceptional circumstances or not at all, or PCI available sometimes, respectively. In contrast, an average proportion of 0.96 (SD 0.03) with STEMI was reported as receiving invasive cardiac management at hospitals with PCI always available. For NSTEMI hospitalisations, there was substantial variation in reporting of invasive cardiac management across all levels of PCI availability: the mean (SD) proportion of people reported as being managed with an invasive cardiac strategy was 0.63 (0.15), 0.71 (0.12) and 0.80 (0.11) for hospitals with PCI available in exceptional circumstances or not available, PCI sometimes available and PCI always available, respectively.

When restricting to people hospitalised with NSTEMI and reduced kidney function, we did not see strong hospital-level associations between the proportion reported receiving invasive cardiac management and the proportion with previous myocardial infarction, prevalent diabetes mellitus or prevalent heart failure (online supplemental figure 2). However, we observed some hospital-level negative association between the proportion receiving invasive cardiac management and the proportion aged ≥80 years.

### Objective 2 – descriptive analyses at the individual level

At the individual level, of the 361 259 unique individuals hospitalised for their first AMI in the study period, 295 019 (82%) had complete covariable data (online supplemental table 5). When restricting to people hospitalised at hospitals with PCI always available, we observed 180 967 unique individuals hospitalised for their first AMI in the study period, with 145 171 (80%) having complete covariable data (online supplemental table 6). For both comparisons, people with missing data had slightly lower prevalences of comorbidities.

We also compared people with missing and non-missing serum creatinine recorded within 24 hours of AMI hospitalisation. People with missing serum creatinine tended to be younger with a lower prevalence of comorbidities (online supplemental tables 7-8).

We focused subsequent analyses on the study population hospitalised for AMI at a hospital with PCI always available and with complete case information. Among these people, 30 834 (21%) had an eGFR corresponding to ranges 3a–5 and/or a chronic renal failure diagnosis (online supplemental table 9). People with ranges 1 and 2 were on average younger (57 (10 SD) and 70 (11 SD) years, respectively) compared with people with ranges 3a, 3b, 4 and 5 (76 (11 SD), 79 (10 SD), 80 (11 SD) and 74 (13 SD)). Comorbidity prevalence tended to be highest among people with eGFR ranges 3b and 4 compared with other eGFR ranges.

Finally, of the 18 924 people with coded renal failure, 12 883 people (68%) had an eGFR corresponding to ranges 3b–5 (online supplemental table 10). Overall, the mean age of this subgroup was 78 years (SD 12) and 38% were female.

### Objective 3 – the association between kidney function and acute myocardial infarction management strategy

The crude proportion of people reported as receiving invasive cardiac management for STEMI at hospitals with PCI available all the time was high (table 2). People with an eGFR range 5 had the lowest crude proportion reported receiving invasive cardiac management (81.1%), while people with an eGFR range 1 had the highest (99.0%). Among people hospitalised for NSTEMI at the same hospitals, the crude proportions reported as treated with invasive cardiac management were lower across all eGFR ranges. People with an eGFR range 1 again had the highest proportion treated with invasive cardiac management (93.6%), while people with eGFR range 4 had the lowest (44.0%).

**Table 2:** Variation in reported invasive versus conservative NSTEMI management between hospitals which offer PCI services all the time in England (n=51) and according to eGFR range at NSTEMI admission)

		n with reported invasive cardiac management (row %)	Row total	Model 1*	Model 2†	Model 3‡	
				OR (95% CI)	OR (95% CI)	OR (95% CI)	APP (95% CI)
STEMI	Missing	5728 (97.3)	5890	–	0.51 (0.25 to 1.05)	0.31 (0.14 to 0.67)	93 (91 to 95)
	eGFR range 1	32 837 (99.0)	33 179	–	2.57 (2.06 to 3.2)	0.89 (0.75 to 1.05)	97 (96 to 98)
	eGFR range 2	32 401 (97.4)	33 280	–	1 (reference)	1 (reference)	97 (96 to 98)
	eGFR range 3a	6778 (93.2)	7273	–	0.37 (0.32 to 0.42)	0.58 (0.51 to 0.67	96 (94 to 97)
	eGFR range 3b	3134 (89.1)	3519	–	0.21 (0.17 to 0.25)	0.42 (0.36 to 0.50)	95 (93 to 96)
	eGFR range 4	886 (81.5)	1087	–	0.11 (0.09 to 0.13)	0.24 (0.2 to 0.29)	92 (89 to 94)
	eGFR range 5	163 (81.1)	201	–	0.11 (0.07 to 0.17)	0.17 (0.11 to 0.27)	89 (85 to 93)
	Coded renal failure	1721 (86.0)	2002	–	0.17 (0.14 to 0.2)	0.32 (0.26 to 0.39)	93 (91 to 95)
	Rho (intracluster coefficient)	–	–	0.27 (0.17 to 0.41)	0.24 (0.17 to 0.42)	0.29 (0.18 to 0.43)	–
NSTEMI	Missing	2397 (86.0)	2788	–	0.86 (0.55 to 1.34)	0.62 (0.43 to 0.89)	80 (76 to 84)
	eGFR range 1	14 882 (93.6)	15 898	–	2.7 (2.17 to 3.35)	0.85 (0.75 to 0.96)	83 (80 to 87)
	eGFR range 2	20 110 (84.5)	23 788	–	1 (reference)	1 (reference)	85 (82 to 88)
	eGFR range 3a	4909 (72.7)	6756	–	0.48 (0.43 to 0.53)	0.75 (0.68 to 0.83)	82 (79 to 86)
	eGFR range 3b	2169 (59.4)	3651	–	0.26 (0.22 to 0.3)	0.48 (0.43 to 0.53)	77 (73 to 81)
	eGFR range 4	520 (44.0)	1180	–	0.13 (0.1 to 0.16)	0.25 (0.20 to 0.31)	68 (63 to 73)
	eGFR range 5	147 (51.6)	285	–	0.17 (0.13 to 0.24)	0.19 (0.13 to 0.26)	64 (57 to 70)
	Coded renal failure	2757 (62.7)	4394	–	0.26 (0.22 to 0.31)	0.4 (0.34 to 0.45)	75 (70 to 79)
	Rho (intracluster coefficient)	–	–	0.26 (0.16 to 0.39)	0.28 (0.17 to 0.42)	0.28 (0.17 to 0.43)	–

To understand the variation in cardiac management across hospitals, we compared three logistic regression models which incrementally accounted for variation across hospitals using centre as a random effect (model 1), additionally eGFR range at AMI admission as a fixed effect (model 2) and additionally several other potential confounders of the association between eGFR range and AMI management strategy (model 3). Of interest is the intracluster coefficient (rho) across these three models. The intracluster coefficient is calculated by dividing the between-cluster variability and the sum of the within-cluster and between-cluster variabilities, meaning it describes the proportion of the variation in the outcome (invasive vs conservative cardiac management) explained by the centre-level variation, after accounting for any other fixed effects included in the model (models 2 and 3).

eGFR ranges (mL/min/1.73 m2): range 1 (≥90), range 2 (60–89), range 3a (45–59), range 3b (30–44), range 4 (15–29) and range 5 (0–14).

* Logistic regression model with cardiology centre as random effect (no fixed effect independent variables).

† Logistic regression model with cardiology centre as random effect and eGFR range as fixed effect.

‡ Logistic regression model with cardiology centre as random effect, eGFR ranges, sex, age, admission year, ethnicity, comorbidities (previous MI, angina, hypertension, hypercholesterolaemia, peripheral vascular disease, COPD, heart failure, type 2 diabetes), co-prescriptions (RASi, beta-blocker, statin).

AMI, acute myocardial infarction; APP, adjusted predicted percent; COPD, chronic obstructive pulmonary disease; eGFR, estimated glomerular filtration rate; MI, myocardial infarction; NSTEMI, non-ST-elevation myocardial infarction; OR, odds ratio; PCI, percutaneous coronary intervention; RASi, renin-angiotensin system inhibitors; STEMI, ST-elevation myocardial infarction.

**Figure 3:**
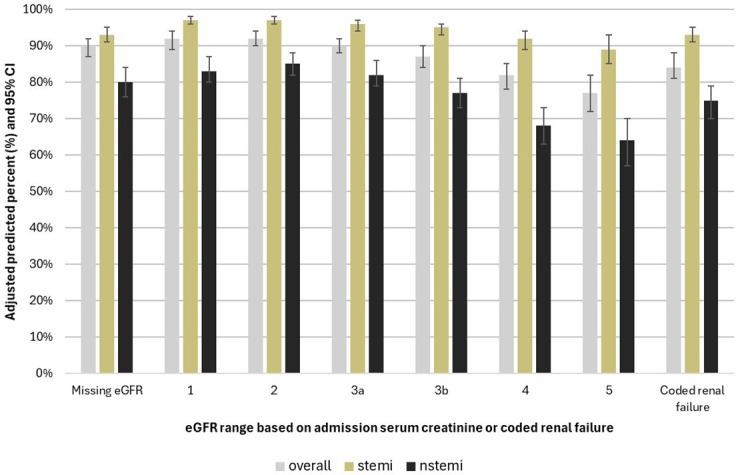
Adjusted predicted percentages of people who receive angiography and/or percutaneous coronary intervention (PCI) by estimated glomerular filtration rate (eGFR) range, overall and stratified by acute myocardial infarction (AMI) subtype. NSTEMI, non-ST-elevation myocardial infarction; STEMI, ST-elevation myocardial infarction.

After adjusting for centre as a random effect and covariables as fixed effects (model 3 in table 2), we found that people hospitalised for both STEMI and NSTEMI with eGFR ranges 3a–5 had lower odds of being reported as receiving invasive versus conservative cardiac management compared with people with eGFR range 2. After converting the adjusted ORs to the adjusted predicted probability scale, we similarly found that people with eGFR ranges 3a to 5 had lower predicted probabilities of being reported as managed invasively versus conservatively for both STEMI and NSTEMI; however, those hospitalised with STEMI had higher predicted percentages of being reported as receiving invasive cardiac management across all eGFR ranges compared with people hospitalised with NSTEMI (table 2 and figure 3). For example, the predicted percent managed invasively for eGFR range 3a was 96% (95% CI 94 to 97) for STEMI and 82% (95% CI 79 to 86) for NSTEMI. For eGFR range 5, the predicted percent managed invasively was 89% (95% CI 85 to 93) for STEMI and 64% (95% CI 57 to 70) for NSTEMI.

The intracluster correlation coefficients from our mixed effect models (table 2) demonstrate a substantial proportion (STEMI: 0.29 (95% CI 0.18 to 0.43); NSTEMI: 0.28 (95% CI 0.17 to 0.43)—all from model 3) of variation in the reported provision of an invasive cardiac strategy between hospitals, even after accounting for individual characteristics and time period.

In a secondary analysis which included all hospitals in the MINAP data (PCI available in exceptional circumstances or not at all, PCI available sometimes and PCI always available), we found similar trends in decreasing odds and predicted percent of people receiving invasive versus conservative cardiac management for people with worsening eGFR range across the three hospital types (online supplemental table 11). People receiving care in hospitals which have PCI always available had higher predicted percentages of people reported as receiving invasive versus conservative cardiac management.

## Discussion

### Summary of findings

We described substantial variation in the proportion of people hospitalised with AMI receiving invasive versus conservative cardiac management across hospitals in England reported in MINAP data. Even after adjustment for individual-level characteristics, there was substantial between-hospital variation in the provision of invasive versus conservative cardiac management. This reported variation was more pronounced among people with NSTEMI and with an admission serum creatinine indicating reduced kidney function.

At the individual level, we observed a relative decrease in the odds of invasive versus conservative cardiac management by worsening kidney function in hospitals with PCI available all the time. However, these differences were less extreme when considered on the absolute probability scale. We observed lower adjusted predicted probabilities of being treated with invasive cardiac treatment with decreasing eGFR range, although the adjusted probability was relatively high across all eGFR levels for STEMI (89%–97%) and NSTMEMI hospitalisations (64%–85%).

Our findings demonstrate that similar individuals, particularly those with reduced kidney function, are likely to be treated differently for NSTEMI dependent on the hospital to which they are admitted. Understanding the factors leading to this variation is difficult using routinely collected audit data. For example, some hospitals/operators may be more prepared to use contrast-sparing methods at angiography and PCI or have greater confidence in taking on complex PCI than others. Furthermore, local policies might influence decisions on which type of hospital an ambulance crew transports patients based on the expected balance of benefits versus risks of an invasive approach. This clinical uncertainty, particularly in people with reduced kidney function who are at increased risk of contrast-induced nephropathy and bleeding, is likely a significant factor contributing to the variation in AMI management we observed in this study.

### Strengths

This study used large and nationally representative data from MINAP, part of the National Institute of Cardiovascular Outcomes Research audit programme.^18 20 28^ The data included granular AMI treatment and covariable information, which enabled us to observe management variation across AMI subtypes and according to levels of kidney function—an important clinical characteristic when considering AMI management strategies. We found similar variation across hospitals in England as a previous study which used the same data source but only covered AMI hospitalisations between 2004 and 2010.^19^ Further, we investigated variation specifically among people with reduced kidney function, a high-risk group which is largely excluded from clinical trials,^7^ leading to ambiguity in nationwide clinical guidelines from NICE.^8^

A previous study investigated individual-level associations between particular comorbidities captured in MINAP, including chronic renal failure, and receipt of optimal guideline-recommended AMI management and found no evidence that people with coded renal failure had lower odds of optimal AMI management.^29^ Our study investigated a broader subgroup of people hospitalised for AMI with reduced kidney function and found that people with coded chronic renal failure had a lower probability of receiving invasive cardiac management compared with people with no evidence of reduced kidney function. Moreover, people with coded renal failure had a higher probability of receiving this treatment compared with people with eGFR ranges 4–5 for both STEMI and NSTEMI hospitalisations. We were unable to distinguish between people with transplanted kidneys, people on dialysis and people with other types of kidney disease within this subgroup, which would help in understanding why people with coded renal failure are more likely to receive invasive cardiac management compared with people with eGFR ranges 4 and 5.

### Limitations

These data were unlinked to other routinely collected health datasets. We know from previous work that AMI case ascertainment is associated with baseline kidney function^27^ and is incomplete with MINAP alone,^27 30 31^ particularly since MINAP focuses on capturing type 1 AMI.^20^ Our study population is therefore only a selection of all AMI hospitalisations in England, namely, those reported in MINAP, making these results vulnerable to collider bias. The substantial variation we observed in these data is likely to reflect true variations in treatment, but also regional variations in AMI pathways and reporting issues (such as non-PCI hospitals failing to report procedures carried out off-site and reporting more type 2 AMI or medically managed AMI in highly co-morbid patients). These reporting issues are highlighted where we observed unrealistically low proportions of people receiving invasive versus conservative cardiac management in hospitals which only offer PCI in exceptional circumstances or not at all. Thus, we are careful to explain that the variation we observed is the reported variation in the MINAP data and may not reflect true variation in AMI care.

We also relied on serum creatinine recorded at the time of AMI hospitalisation to determine baseline kidney function and eGFR range. Thus, misclassification of reduced kidney function is likely. We assumed that people with a missing serum creatinine result did not have reduced kidney function. While we know that assuming missing eGFR in primary care electronic health record data yields accurate prevalence estimates for reduced kidney function,^23^ this may not necessarily be true in secondary care data. Further, since many patients may experience acute declines in kidney function at the time of AMI hospitalisation due to intercurrent illness, or as previous work demonstrated, random error in the estimation of eGFR is defined in secondary versus primary care.^27^ Yet it is likely to be the creatinine level on hospitalisation (or soon after) that is taken into account by the clinician referring for (or accepting for) angiography, making these results relevant to healthcare decision-makers.

For all other missing covariable data, we used a complete case analysis approach. People with missing covariable data tended to have lower recorded prevalences of comorbidities. By excluding these people, we may have overestimated variation in AMI management, since healthier people tend to receive invasive vs conservative cardiac management.

We adjusted for measured confounders in our multivariable models quantifying the association between reduced kidney function and AMI management strategy. However, our findings are likely impacted by residual confounding by factors such as frailty, which influence both the degree of kidney impairment and AMI management strategies people receive in hospital.

Finally, we did not incorporate the potential time between the exposure measurement (serum creatinine laboratory test) and AMI management in the study design and analysis, since the timings of the exposure and outcomes were incompletely described in these data.

### Future research

The comparative and cost-effectiveness of invasive versus conservative management strategies for people with impaired kidney function hospitalised for NSTEMI is uncertain. The treatment variation we described in this study should be further explored in linked secondary care data to understand if this variation can be exploited in a natural experiment, comparing invasive versus conservative NSTEMI management strategies in people with reduced kidney function and/or CKD for important outcomes like mortality. Linking these audit data with other routinely collected health data is important to improve AMI case ascertainment and reliably estimate baseline kidney function.

## Conclusions

We highlighted substantial reported AMI management variation across hospitals in England. This variation is particularly pronounced among people hospitalised for NSTEMI and with reduced kidney function. Further research is needed to understand the comparative effectiveness of invasive versus conservative NSTEMI management strategies among people with reduced kidney function to improve outcomes for patients.

## Supplementary material

10.1136/bmjopen-2024-096991 online supplemental file 1

## Data Availability

Data may be obtained from a third party and are not publicly available.
