# Supplementary material for: Variation in acute myocardial infarction management by kidney function across hospitals in England: a cross-sectional study using the Myocardial Ischaemia National Audit Project (MINAP)
Source: BMJ Open. 2025 May 16;15(5):e096991. doi: 10.1136/bmjopen-2024-096991 (PMC12086913; doi:10.1136/bmjopen-2024-096991)

**Supplementary materials**

Variation in acute myocardial infarction management by kidney function across hospitals in England: a cross-sectional study using the Myocardial Ischaemia National Audit Project (MINAP)

Contents

[**SUPPLEMENTARY TABLES** 3](#_Toc193128347)

[**Supplementary table 1.** Variables used to define outcomes* 4](#_Toc193128348)

[**Supplementary table 2.** CALIBER definition of AMI subtypes (STEMI, NSTEMI) using MINAP data* 5](#_Toc193128349)

[**Supplementary table 3.** Categorisation of hospitals in England by cardiac intervention (percutaneous coronary intervention (PCI)) service availability, 2018-19 6](#_Toc193128350)

[**Supplementary table 4.** Transfers between hospitals during the same AMI event in the hospital-level and individual-level (complete cases) analysis, all hospitals (N=209) 9](#_Toc193128351)

[**Supplementary table 5.** Description of complete (included in the complete case analysis) and incomplete cases (excluded from the complete case analysis) 10](#_Toc193128352)

[**Supplementary table 6.** Description of complete (included in the complete case analysis) and incomplete cases (excluded from the complete case analysis) who are hospitalised at hospitals providing percutaneous coronary intervention (PCI) all the time 12](#_Toc193128353)

[**Supplementary table 7.** Description of people with missing versus non-missing serum creatinine (SCr) value 14](#_Toc193128354)

[**Supplementary table 8.** Description of people with missing versus non-missing serum creatinine (SCr) value hospitalised where percutaneous coronary intervention services are available all the time 16](#_Toc193128355)

[**Supplementary table 9.** Baseline characteristics at first AMI hospitalisation during the study period between 2014-2019 among complete cases, in hospitals with PCI services available all the time (N=51) 18](#_Toc193128356)

[**Supplementary table 10.** Description of people with coded chronic renal failure, stratified by the eGFR stage corresponding to the SCr measured within 24 hours of hospitalisation (all hospitals, N=209) 20](#_Toc193128357)

[**Supplementary table 11.** Association between eGFR stage and invasive versus conservative cardiac management in the overall study population (STEMI and NSTEMI hospitalisations), stratified by centres’ PCI availability 22](#_Toc193128358)

[**SUPPLEMENTARY FIGURES** 25](#_Toc193128359)

[**Supplementary figure 1.** Variation in AMI treatment (angiography and/or PCI) at the centre-level (N=209 centres total), for (a) STEMI and (b) NSTEMI. 26](#_Toc193128360)

[**Supplementary figure 2.** Centre-level variation in the proportion receiving invasive cardiac treatment versus the proportion with clinically important characteristics (aged ≥80 years, previous myocardial infarction, prevalent diabetes mellitus, and prevalent heart failure) 27](#_Toc193128361)

# **SUPPLEMENTARY TABLES**

### **Supplementary table 1.** Variables used to define outcomes*

| **Process of care** | **MINAP definition** |
| --- | --- |
| Angiography | Angiography defined using data from the following variables:   - Interventional hospital procedure - Coronary angiography - Why no angiography - Procedure performed - Additional reperfusion treatment - Why no intervention - Coronary intervention |
| Percutaneous coronary intervention (PCI) | PCI defined using data from the following variables:   - Why no intervention - Coronary intervention - Interventional hospital procedure - Procedure performed at admission - Initial reperfusion treatment - Additional reperfusion treatment |
| Coronary artery bypass graft (CABG) | CABG defined using data from the following variables:   - Why no intervention - Coronary intervention - Interventional hospital procedure |

*Table adapted from previous publications:

Bidulka P, Scott J, Taylor DM, et al. Impact of chronic kidney disease on case ascertainment for hospitalised acute myocardial infarction: an English cohort study. *BMJ Open* 2022;12:e057909. doi: 10.1136/bmjopen-2021-057909

Scott, J., Bidulka, P., Taylor, D.M. et al. Management and outcomes of myocardial infarction in people with impaired kidney function in England. *BMC Nephrol* 24, 325 (2023). https://doi.org/10.1186/s12882-023-03377-x

**Supplementary table 2.** CALIBER definition of AMI subtypes (STEMI, NSTEMI) using MINAP data*

|  | **MINAP variable** | | |
| --- | --- | --- | --- |
| **AMI subtype** | **Discharge diagnosis** | **Markers elevated?** | **ECG result** |
| Other | Threatened MI, Chest pain uncertain cause, MI unconfirmed, other diagnosis | - | - |
| STEMI | STEMI | Raised or missing | ST elevation, LBBB, or ST elevation |
|  | NSTEMI/Troponin positive ACS | Raised or missing | ST elevation |
|  | ACS troponin negative | Raised | ST elevation |
|  | ACS troponin unspecified | Raised | ST elevation |
| NSTEMI | NSTEMI/Troponin positive ACS | Raised or missing | ST depression, T wave changes only, other abnormality, Normal ECG, or LBBB |
|  | ACS troponin negative | Raised | LBBB, ST depression, T wave changes only, Other abnormality, normal ECG, or missing |
|  | ACS troponin unspecified | Raised | LBBB, ST depression, T wave changes only, Other abnormality, normal ECG, or missing |
| Unstable angina | *Any remaining hospitalisations not assigned as STEMI, NSTEMI, or other diagnosis | | |
|  |  |  |  |
| ACS: acute coronary syndrome, AMI: acute myocardial infarction, ECG: electrocardiogram, LBBB: left bundle branch block, MI: myocardial infarction, MINAP: Myocardial Ischaemia National Audit Project, NSTEMI: Non ST-elevation myocardial infarction, STEMI: ST-elevation myocardial infarction | | | |

*Table adapted from previous work: Bidulka P, Scott J, Taylor DM, et al. Impact of chronic kidney disease on case ascertainment for hospitalised acute myocardial infarction: an English cohort study. *BMJ Open* 2022;12:e057909. doi: 10.1136/bmjopen-2021-057909

### **Supplementary table 3.** Categorisation of hospitals in England by cardiac intervention (percutaneous coronary intervention (PCI)) service availability, 2018-19

| **PCI available all the time (N=58)** | | **PCI available sometimes (N=59)** | |
| --- | --- | --- | --- |
| **PCI descriptor** | **Hospital centre name (acronym)** | **PCI descriptor** | **Hospital centre name (acronym)** |
| PPCI 24/7 365 days | Altnagelvin Hospital (ALT) | PPCI hybrid working hours | Wycombe Hospital (AMG) |
|  | Essex Cardiothoracic Centre (BAS) |  | Royal United Hospital Bath (BAT) |
|  | Liverpool Cardiothoracic Centre (BHL) |  | Cheltenham General Hospital (CHG) |
|  | Royal Berkshire and Battle Hospital (BHR) |  | Wiltshire Cardiac Centre (PMS) |
|  | Royal Bournemouth Hospital (BOU) |  | Salisbury District Hospital (SAL) |
|  | Bristol Royal Infirmary (BRI) |  | Dorset County Hospital (WDH) |
|  | Castle Hill Hospital (CHH) | PPCI hybrid 24/7 | Conquest Hospital (CGH) |
|  | Nottingham City Hospital (CHN) |  | Eastbourne Hospital (DGE) |
|  | North Wales Cardiac Centre (CLW) |  | Manchester Royal Infirmary (MRI) |
|  | Cumberland Infirmary (CMI) |  | Wythenshawe Hospital (WYT) |
|  | Royal Derby Hospital (DER) | Self-presenters and in-patients 24/7 | Sunderland Royal Hospital (SUN) |
|  | Birmingham City Hospital (DUD) | Self-presenters and in-patients working hours | Royal Blackburn Hospital (BLA) |
|  | Birmingham Heartlands Hospital (EBH) |  | Southmead Hospital Bristol (BSM) |
|  | Edinburgh Heart Centre (ERI) |  | Craigavon Hospital (CRG) |
|  | Freeman Hospital (FRE) |  | Darent Valley Hospital (DVH) |
|  | Frimley Park Hospital (FRM) |  | Ealing Hospital (EAL) |
|  | St George's Hospital (GEO) |  | East Surrey Hospital (ESU) |
|  | Golden Jubilee National Hospital (GJH) |  | Royal Gwent Hospital (GWE) |
|  | Glenfield Hospital (GRL) |  | Queen Elizabeth Hospital Woolwich (GWH) |
|  | Hairmyres Hospital (HAI) |  | Tunbridge Wells Hospital (KSX) |
|  | Hammersmith Hospital (HAM) |  | Luton and Dunstable University Hospital (LDH) |
|  | Royal Brompton Hospital and Harefield NHS Trust, Harefield site (HH) |  | Croydon University Hospital (MAY) |
|  | Kings College Hospital (KCH) |  | Medway Maritime Hospital (MDW) |
|  | Kettering General Hospital (KGH) |  | Northwick Park Hospital (NPH) |
|  | Yorkshire Heart Centre (LGI) |  | Northampton General Hospital (NTH) |
|  | Lincoln County Hospital (LIN) |  | Raigmore Hospital (RAI) |
|  | Lister Hospital (LIS) |  | Watford General Hospital (WAT) |
|  | Morriston Hospital (MOR) |  | West Middlesex University Hospital (WMU) |
|  | Musgrove Park Hospital (MPH) |  | Worthing Hospital (WRG) |
|  | New Cross Hospital (NCR) | PPCI either in exceptional circumstances only or never | Royal Albert Edward Infirmary (AEI) |
|  | Northern General Hospital (NGS) |  | BMI Alexandra Hospital (AHM) |
|  | Basingstoke and North Hampshire Hospital (NHH) |  | St Anthony's Hospital (ANT) |
|  | Ninewells Hospital (NIN) |  | Bedford Hospital (BED) |
|  | Norfolk and Norwich University Hospital (NOR) |  | BMI Meriden Hospital (BMI) |
|  | Papworth Hospital (PAP) |  | Bradford Royal Infirmary (BRD) |
|  | Derriford Hospital, Southwest Cardiothoracic Centre (PLY) |  | Acute Pennine Trust Fairfield (BRY) |
|  | Queen Alexandra Hospital (QAP) |  | Cromwell Hospital (CRO) |
|  | Queen Elizabeth Hospital, Birmingham (QEB) |  | Duchy Hospital (DUC) |
|  | John Radcliffe Hospital (RAD) |  | Spire Hospital Bristol (GHB) |
|  | Royal Cornwall Hospital (RCH) |  | Spire Hospital Hull and East Riding (HBP) |
|  | Royal Devon & Exeter Hospital (RDE) |  | Wellington Hospital (HHW) |
|  | Royal Free Hospital (RFH) |  | Harley Street Clinic (HSC) |
|  | Aberdeen Royal Infirmary (RIA) |  | London Independent Hospital (IND) |
|  | Royal Sussex County Hospital (RSC) |  | Ipswich Hospital (IPS) |
|  | Royal Victoria Hospital (RVB) |  | Kent Institute of Medicine & Surgery (KIM) |
|  | Barts Health Centre, St Bartholomew’s Hospital (SBH) |  | Kings Mill Hospital (KMH) |
|  | James Cook University Hospital (SCM) |  | London Bridge Hospital (LBH) |
|  | Southampton General Hospital (SGH) |  | Leeds Nuffield Hospital (LNH) |
|  | St Peter's Hospital (SPH) |  | Manor Hospital Oxford (MHO) |
|  | St Thomas' Hospital (STH) |  | Nuffield Health Bournemouth Hospital (NBO) |
|  | University Hospital of North Staffordshire (STO) |  | BMI Priory Hospital (PHB) |
|  | Torbay Hospital (TOR) |  | BMI Park Hospital (PHN) |
|  | University Hospital of Wales (UHW) |  | Pinderfields General Hospital (PIN) |
|  | Blackpool Victoria Hospital (VIC) |  | Ross Hall Hospital (RHH) |
|  | University Hospital Coventry (WAL) |  | Calderdale Royal Hospital (RHI) |
|  | Wexham Park Hospital (WEX) |  | Scunthorpe General Hospital (SCU) |
|  | William Harvey Hospital (WHH) |  | Spire Cardiff Hospital (SPC) |
|  | Worcester Royal Hospital (WRC) |  | Spire Shawfair Park Hospital (SSP) |
|  |  |  | York District General Hospital (YDH) |

*N=90 hospitals not listed here but included in the study dataset were categorised as PCI available in never available

### **Supplementary table 4.** Transfers between hospitals during the same AMI event in the hospital-level and individual-level (complete cases) analysis, all hospitals (N=209)

| **Number of transfers during AMI event** | **Centre-level analysis**  **Number of patients, n (% of total patients)** | **Individual-level analysis (complete cases)**  **Number of patients, n (% of total patients)** |
| --- | --- | --- |
| **0** | 334,908 (93) | 270, 382 (92) |
| **1** | 26,256 (7) | 24,548 (8) |
| **2** | 92 (0) | 86 (0) |
| **3** | <5 (0) | <5 (0) |
| **Total** | 361,259 (100) | 295,019 (100) |

AMI: acute myocardial infarction

### **Supplementary table 5.** Description of complete (included in the complete case analysis) and incomplete cases (excluded from the complete case analysis)

|  | **Total** | **Complete cases** | **Incomplete cases** |
| --- | --- | --- | --- |
| **N= (row %)** | **361,259 (100)** | **295,019 (100)** | **66,240 (100)** |
| **Sex** |  |  |  |
| Female | 117,415 (33) | 95,792 (32) | 21,623 (34) |
| Male | 241,654 (67) | 199,227 (68) | 42,427 (66) |
| Sex missing | 2,190 (1) | 0 (0) | 2,190 (3) |
| **Age in years, mean (SD)** | 69 (14) | 69 (14) | 70 (14) |
| **Age category in years (%)** |  |  |  |
| <50 | 30,796 (9) | 25,578 (9) | 5,218 (8) |
| 50-59 | 64,483 (18) | 53,526 (18) | 10,957 (17) |
| 60-69 | 82,030 (23) | 68,009 (23) | 14,021 (21) |
| 70-79 | 88,494 (25) | 72,376 (25) | 16,118 (24) |
| 80+ | 95,213 (26) | 75,530 (26) | 19,683 (30) |
| Missing | 243 (0) | 0 (0) | 243 (0) |
| **Ethnicity** |  |  |  |
| White | 282,991 (90) | 265,373 (90) | 17,618 (91) |
| Black | 3,309 (1) | 3,071 (1) | 238 (1) |
| Asian | 20,479 (7) | 19,384 (7) | 1,095 (6) |
| Mixed/other | 7,561 (2) | 7,191 (2) | 370 (2) |
| Missing | 46,919 (13) | 0 (0) | 46,919 (71) |
| **eGFR range** |  |  |  |
| Missing eGFR | 20,584 (6) | 13,346 (5) | 7,238 (11) |
| 1 | 102,624 (28) | 86,531 (29) | 16,093 (24) |
| 2 | 141,043 (39) | 117,209 (40) | 23,834 (36) |
| 3a | 40,499 (11) | 32,815 (11) | 7,684 (12) |
| 3b | 23,553 (7) | 18,625 (6) | 4,928 (7) |
| 4 | 8,155 (2) | 6,270 (2) | 1,885 (3) |
| 5 | 1,573 (0) | 1,163 (0) | 410 (1) |
| Coded renal failure | 23,228 (6) | 19,060 (6) | 4,168 (6) |
| **Comorbidities** |  |  |  |
| Angina | 67,981 (19) | 57,928 (20) | 10,053 (15) |
| Cerebrovascular disease | 26,746 (7) | 22,211 (8) | 4,535 (7) |
| COPD | 53,959 (15) | 45,995 (16) | 7,964 (12) |
| Diabetes mellitus | 86,283 (24) | 71,273 (24) | 15,010 (23) |
| Heart failure | 20,201 (6) | 16,735 (6) | 3,466 (5) |
| Hypercholesterolaemia | 106,833 (30) | 92,028 (31) | 14,805 (22) |
| Hypertension | 171,975 (48) | 144,372 (49) | 27,603 (42) |
| Previous MI | 66,410 (18) | 56,207 (19) | 10,203 (15) |
| Peripheral vascular disease | 15,015 (4) | 12,853 (4) | 2,162 (3) |
| Kidney failure | 23,228 (6) | 19,060 (6) | 4,168 (6) |
| **Co-prescriptions** |  |  |  |
| Beta-blocker | 86,220 (24) | 72,887 (25) | 13,333 (20) |
| RASi | 111,493 (31) | 94,459 (32) | 17,034 (26) |
| Statin | 136,181 (38) | 114,264 (39) | 21,917 (33) |
| **Smoking status** |  |  |  |
| Non-smoker | 137,251 (40) | 119,442 (40) | 17,809 (40) |
| Ex-smoker | 111,258 (33) | 96,749 (33) | 14,509 (33) |
| Current smoker | 90,747 (27) | 78,828 (27) | 11,919 (27) |
| Missing | 22,003 (6) | 0 (0) | 22,003 (33) |

eGFR ranges (mL/min/1.73m^2^): range 1 (≥90), range 2 (60-89), range 3a (45-59), range 3b (30-44), range 4 (15-29), and range 5 (0-14)

### **Supplementary table 6.** Description of complete (included in the complete case analysis) and incomplete cases (excluded from the complete case analysis) who are hospitalised at hospitals providing percutaneous coronary intervention (PCI) all the time

|  | **Total** | **Complete cases** | **Incomplete cases** |
| --- | --- | --- | --- |
| **N= (row %)** | **180,967 (100)** | **145,171 (100)** | **35,796 (100)** |
| **Sex** |  |  |  |
| Female | 127,311 (71) | 103,535 (71) | 23,776 (70) |
| Male | 52,057 (29) | 41,636 (29) | 10,421 (30) |
| Sex missing | 1,599 (1) | 0 (0) | 1,599 (4) |
| **Age in years, mean (SD)** | 67 (13) | 67 (13) | 68 (14) |
| **Age category in years (%)** |  |  |  |
| <50 | 18,417 (10) | 15,086 (10) | 3,331 (9) |
| 50-59 | 37,451 (21) | 30,608 (21) | 6,843 (19) |
| 60-69 | 44,749 (25) | 36,349 (25) | 8,400 (23) |
| 70-79 | 43,117 (24) | 34,444 (24) | 8,673 (24) |
| 80+ | 37,212 (21) | 28,684 (20) | 8,528 (24) |
| Missing | 21 (0) | 0 (0) | 21 (0) |
| **Ethnicity** |  |  |  |
| White | 138,634 (89) | 129,641 (89) | 8,993 (91) |
| Black | 1,635 (1) | 1,525 (1) | 110 (1) |
| Asian | 11,068 (7) | 10,450 (7) | 618 (6) |
| Mixed/other | 3,712 (2) | 3,555 (2) | 157 (2) |
| Missing | 25,918 (14) | 0 (0) | 25,918 (72) |
| **eGFR range** |  |  |  |
| Missing eGFR | 13,780 (8) | 8,678 (6) | 5,102 (14) |
| 1 | 58,938 (33) | 49,077 (34) | 9,861 (28) |
| 2 | 69,534 (38) | 57,068 (39) | 12,466 (35) |
| 3a | 17,675 (10) | 14,029 (10) | 3,646 (10) |
| 3b | 9,361 (5) | 7,170 (5) | 2,191 (6) |
| 4 | 3,054 (2) | 2,267 (2) | 787 (2) |
| 5 | 690 (0) | 486 (0) | 204 (1) |
| Coded renal failure | 7,935 (4) | 6,396 (4) | 1,539 (4) |
| **Comorbidities** |  |  |  |
| Angina | 24,377 (13) | 20,409 (14) | 3,968 (11) |
| Cerebrovascular disease | 10,149 (6) | 8,304 (6) | 1,845 (5) |
| COPD | 22,137 (12) | 18,540 (13) | 3,597 (10) |
| Diabetes mellitus | 38,312 (21) | 31,246 (22) | 7,066 (20) |
| Heart failure | 6,453 (4) | 5,263 (4) | 1,190 (3) |
| Hypercholesterolaemia | 51,768 (29) | 44,511 (31) | 7,257 (20) |
| Hypertension | 79,335 (44) | 65,833 (45) | 13,502 (38) |
| Previous MI | 26,054 (14) | 21,766 (15) | 4,288 (12) |
| Peripheral vascular disease | 6,495 (4) | 5,459 (4) | 1,036 (3) |
| Kidney failure | 7,935 (4) | 6,396 (4) | 1,539 (4) |
| **Co-prescriptions** |  |  |  |
| Beta-blocker | 33,665 (19) | 28,309 (20) | 5,356 (15) |
| RASi | 45,499 (25) | 38,256 (26) | 7,243 (20) |
| Statin | 56,624 (31) | 47,177 (32) | 9,447 (26) |
| **Smoking status** |  |  |  |
| Non-smoker | 65,792 (39) | 56,379 (39) | 9,413 (39) |
| Ex-smoker | 51,157 (30) | 43,623 (30) | 7,534 (31) |
| Current smoker | 52,519 (31) | 45,169 (31) | 7,350 (30) |
| Missing | 11,499 (6) | 0 (0) | 11,499 (32) |

eGFR ranges (mL/min/1.73m^2^): range 1 (≥90), range 2 (60-89), range 3a (45-59), range 3b (30-44), range 4 (15-29), and range 5 (0-14)

### **Supplementary table 7.** Description of people with missing versus non-missing serum creatinine (SCr) value

|  | **Total** | **Non-missing SCr** | **Missing SCr** |
| --- | --- | --- | --- |
| **N= (row %)** | **361,259 (100)** | **340,675 (100)** | **20,584 (100)** |
| **Sex** |  |  |  |
| Female | 241,654 (67) | 228,493 (67) | 13,161 (71) |
| Male | 117,415 (33) | 112,100 (33) | 5,315 (29) |
| Sex missing | 2,190 (1) | 82 (0) | 2,108 (10) |
| **Age in years, mean (SD)** | 69 (14) | 69 (14) | 64 (16) |
| **Age category in years (%)** |  |  |  |
| <50 | 30,796 (9) | 26,927 (8) | 3,869 (19) |
| 50-59 | 64,483 (18) | 60,425 (18) | 4,058 (20) |
| 60-69 | 82,030 (23) | 77,549 (23) | 4,481 (22) |
| 70-79 | 88,494 (25) | 84,312 (25) | 4,182 (21) |
| 80+ | 95,213 (26) | 91,445 (27) | 3,768 (19) |
| Missing | 243 (0) | 17 (0) | 226 (1) |
| **Ethnicity** |  |  |  |
| White | 282,991 (90) | 267,651 (90) | 15,340 (87) |
| Black | 3,309 (1) | 3,106 (1) | 203 (1) |
| Asian | 20,479 (7) | 18,876 (6) | 1,603 (9) |
| Mixed/other | 7,561 (2) | 7,045 (2) | 516 (3) |
| Missing | 46,919 (13) | 43,997 (13) | 2,922 (14) |
| **eGFR range** |  |  |  |
| Missing eGFR | 20,584 (6) | 0 (0) | 20,584 (100) |
| 1 | 102,624 (28) | 102,624 (30) | 0 (0) |
| 2 | 141,043 (39) | 141,043 (41) | 0 (0) |
| 3a | 40,499 (11) | 40,499 (12) | 0 (0) |
| 3b | 23,553 (7) | 23,553 (7) | 0 (0) |
| 4 | 8,155 (2) | 8,155 (2) | 0 (0) |
| 5 | 1,573 (0) | 1,573 (0) | 0 (0) |
| Coded renal failure | 23,228 (6) | 23,228 (7) | 0 (0) |
| **Comorbidities** |  |  |  |
| Angina | 67,981 (19) | 65,285 (19) | 2,696 (13) |
| Cerebrovascular disease | 26,746 (7) | 25,785 (8) | 961 (5) |
| COPD | 53,959 (15) | 51,775 (15) | 2,184 (11) |
| Diabetes mellitus | 86,283 (24) | 82,124 (24) | 4,159 (20) |
| Heart failure | 20,201 (6) | 19,577 (6) | 624 (3) |
| Hypercholesterolaemia | 106,833 (30) | 101,576 (30) | 5,257 (26) |
| Hypertension | 171,975 (48) | 164,329 (48) | 7,646 (37) |
| Previous MI | 66,410 (18) | 63,628 (19) | 2,782 (14) |
| Peripheral vascular disease | 15,015 (4) | 14,471 (4) | 544 (3) |
| Kidney failure | 23,228 (6) | 23,228 (7) | 0 (0) |
| **Co-prescriptions** |  |  |  |
| Beta-blocker | 86,220 (24) | 83,384 (24) | 2,836 (14) |
| RASi | 111,493 (31) | 107,863 (32) | 3,630 (18) |
| Statin | 136,181 (38) | 130,939 (38) | 5,242 (25) |
| **Smoking status** |  |  |  |
| Non-smoker | 137,251 (40) | 130,623 (41) | 6,628 (38) |
| Ex-smoker | 111,258 (33) | 106,457 (33) | 4,801 (27) |
| Current smoker | 90,747 (27) | 84,687 (26) | 6,060 (35) |
| Missing | 22,003 (6) | 18,908 (6) | 3,095 (15) |

eGFR ranges (mL/min/1.73m^2^): range 1 (≥90), range 2 (60-89), range 3a (45-59), range 3b (30-44), range 4 (15-29), and range 5 (0-14)

### **Supplementary table 8.** Description of people with missing versus non-missing serum creatinine (SCr) value hospitalised where percutaneous coronary intervention services are available all the time

|  | **Total** | **Non-missing SCr** | **Missing SCr** |
| --- | --- | --- | --- |
| **N= (row %)** | **180,967 (100)** | **167,187 (100)** | **13,780 (100)** |
| **Sex** |  |  |  |
| Female | 127,311 (71) | 118,419 (71) | 8,892 (73) |
| Male | 52,057 (29) | 48,728 (29) | 3,329 (27) |
| Sex missing | 1,599 (1) | 40 (0) | 1,559 (11) |
| **Age in years, mean (SD)** | 67 (13) | 67 (13) | 63 (15) |
| **Age category in years (%)** |  |  |  |
| <50 | 18,417 (10) | 15,650 (9) | 2,767 (20) |
| 50-59 | 37,451 (21) | 34,437 (21) | 3,014 (22) |
| 60-69 | 44,749 (25) | 41,569 (25) | 3,180 (23) |
| 70-79 | 43,117 (24) | 40,370 (24) | 2,747 (20) |
| 80+ | 37,212 (21) | 35,159 (21) | 2,053 (15) |
| Missing | 21 (0) | 2 (0) | 19 (0) |
| **Ethnicity** |  |  |  |
| White | 138,634 (89) | 128,497 (90) | 10,137 (86) |
| Black | 1,635 (1) | 1,518 (1) | 117 (1) |
| Asian | 11,068 (7) | 9,958 (7) | 1,110 (9) |
| Mixed/other | 3,712 (2) | 3,355 (2) | 357 (3) |
| Missing | 25,918 (14) | 23,859 (14) | 2,059 (15) |
| **eGFR range** |  |  |  |
| Missing eGFR | 13,780 (8) | 0 (0) | 13,780 (100) |
| 1 | 58,938 (33) | 58,938 (35) | 0 (0) |
| 2 | 69,534 (38) | 69,534 (42) | 0 (0) |
| 3a | 17,675 (10) | 17,675 (11) | 0 (0) |
| 3b | 9,361 (5) | 9,361 (6) | 0 (0) |
| 4 | 3,054 (2) | 3,054 (2) | 0 (0) |
| 5 | 690 (0) | 690 (0) | 0 (0) |
| Coded renal failure | 7,935 (4) | 7,935 (5) | 0 (0) |
| **Comorbidities** |  |  |  |
| Angina | 24,377 (13) | 22,862 (14) | 1,515 (11) |
| Cerebrovascular disease | 10,149 (6) | 9,657 (6) | 492 (4) |
| COPD | 22,137 (12) | 20,850 (12) | 1,287 (9) |
| Diabetes mellitus | 38,312 (21) | 35,828 (21) | 2,484 (18) |
| Heart failure | 6,453 (4) | 6,116 (4) | 337 (2) |
| Hypercholesterolaemia | 51,768 (29) | 48,430 (29) | 3,338 (24) |
| Hypertension | 79,335 (44) | 74,642 (45) | 4,693 (34) |
| Previous MI | 26,054 (14) | 24,415 (15) | 1,639 (12) |
| Peripheral vascular disease | 6,495 (4) | 6,133 (4) | 362 (3) |
| Kidney failure | 7,935 (4) | 7,935 (5) | 0 (0) |
| **Co-prescriptions** |  |  |  |
| Beta-blocker | 33,665 (19) | 32,113 (19) | 1,552 (11) |
| RASi | 45,499 (25) | 43,486 (26) | 2,013 (15) |
| Statin | 56,624 (31) | 53,605 (32) | 3,019 (22) |
| **Smoking status** |  |  |  |
| Non-smoker | 65,792 (39) | 61,538 (39) | 4,254 (37) |
| Ex-smoker | 51,157 (30) | 48,133 (30) | 3,024 (26) |
| Current smoker | 52,519 (31) | 48,179 (31) | 4,340 (37) |
| Missing | 11,499 (6) | 9,337 (6) | 2,162 (16) |

eGFR ranges (mL/min/1.73m^2^): range 1 (≥90), range 2 (60-89), range 3a (45-59), range 3b (30-44), range 4 (15-29), and range 5 (0-14)

### **Supplementary table 9.** Baseline characteristics at first AMI hospitalisation during the study period between 2014-2019 among complete cases, in hospitals with PCI services available all the time (N=51)

|  | **Total** | **Missing eGFR*** | **Range 1** | **Range 2** | **Range 3a** | **Range 3b** | **Range 4** | **Range 5** | **Coded chronic renal failure**** |
| --- | --- | --- | --- | --- | --- | --- | --- | --- | --- |
| **N=** | **145,171** | **8,678** | **49,077** | **57,068** | **14,029** | **7,170** | **2,267** | **486** | **6,396** |
| **Female** | 41,636 (29) | 2,298 (26) | 10,290 (21) | 16,942 (30) | 5,376 (38) | 3,173 (44) | 1,119 (49) | 209 (43) | 2,229 (35) |
| **Age in years, mean (SD)** | 67 (13) | 62 (15) | 57 (10) | 70 (11) | 76 (11) | 79 (10) | 80 (11) | 74 (13) | 76 (12) |
| **Age category in years (%)** |  |  |  |  |  |  |  |  |  |
| <50 | 15,086 (10) | 1,991 (23) | 10,210 (21) | 2,309 (4) | 238 (2) | 93 (1) | 34 (1) | 17 (3) | 194 (3) |
| 50-59 | 30,608 (21) | 1,885 (22) | 18,784 (38) | 8,213 (14) | 808 (6) | 245 (3) | 92 (4) | 51 (10) | 530 (8) |
| 60-69 | 36,349 (25) | 1,948 (22) | 15,149 (31) | 14,906 (26) | 2,244 (16) | 810 (11) | 208 (9) | 98 (20) | 986 (15) |
| 70-79 | 34,444 (24) | 1,640 (19) | 4,445 (9) | 19,069 (33) | 4,648 (33) | 2,037 (28) | 562 (25) | 153 (31) | 1,890 (30) |
| 80+ | 28,684 (20) | 1,214 (14) | 489 (1) | 12,571 (22) | 6,091 (43) | 3,985 (56) | 1,371 (60) | 167 (34) | 2,796 (44) |
| **Ethnicity** |  |  |  |  |  |  |  |  |  |
| White | 129,641 (89) | 7,371 (85) | 43,149 (88) | 52,110 (91) | 12,787 (91) | 6,506 (91) | 2,038 (90) | 386 (79) | 5,294 (83) |
| Black | 1,525 (1) | 96 (1) | 427 (1) | 522 (1) | 165 (1) | 90 (1) | 30 (1) | 15 (3) | 180 (3) |
| Asian | 10,450 (7) | 916 (11) | 4,006 (8) | 3,326 (6) | 799 (6) | 431 (6) | 150 (7) | 75 (15) | 747 (12) |
| Mixed/other | 3,555 (2) | 295 (3) | 1,495 (3) | 1,110 (2) | 278 (2) | 143 (2) | 49 (2) | 10 (2) | 175 (3) |
| **Comorbidities** |  |  |  |  |  |  |  |  |  |
| Angina | 20,409 (14) | 1,069 (12) | 4,278 (9) | 8,160 (14) | 2,676 (19) | 1,531 (21) | 445 (20) | 77 (16) | 2,173 (34) |
| Cerebrovascular disease | 8,304 (6) | 322 (4) | 1,319 (3) | 3,307 (6) | 1,278 (9) | 731 (10) | 234 (10) | 35 (7) | 1,078 (17) |
| COPD | 18,540 (13) | 956 (11) | 5,371 (11) | 7,453 (13) | 1,989 (14) | 1,101 (15) | 319 (14) | 54 (11) | 1,297 (20) |
| Diabetes mellitus | 31,246 (22) | 1,704 (20) | 8,316 (17) | 10,700 (19) | 3,786 (27) | 2,438 (34) | 940 (41) | 215 (44) | 3,147 (49) |
| Heart failure | 5,263 (4) | 210 (2) | 606 (1) | 1,645 (3) | 770 (5) | 599 (8) | 231 (10) | 30 (6) | 1,172 (18) |
| Hypercholesterolaemia | 44,511 (31) | 2,260 (26) | 14,361 (29) | 17,923 (31) | 4,473 (32) | 2,242 (31) | 629 (28) | 127 (26) | 2,496 (39) |
| Hypertension | 65,833 (45) | 3,097 (36) | 17,466 (36) | 26,733 (47) | 7,820 (56) | 4,301 (60) | 1,337 (59) | 255 (52) | 4,824 (75) |
| Previous MI | 21,766 (15) | 1,094 (13) | 5,168 (11) | 8,330 (15) | 2,858 (20) | 1,628 (23) | 487 (21) | 92 (19) | 2,109 (33) |
| Peripheral vascular disease | 5,459 (4) | 251 (3) | 1,045 (2) | 1,921 (3) | 743 (5) | 399 (6) | 142 (6) | 23 (5) | 935 (15) |
| Kidney failure | 6,396 (4) | 0 (0) | 0 (0) | 0 (0) | 0 (0) | 0 (0) | 0 (0) | 0 (0) | 6,396 (100) |
| **Co-prescriptions** |  |  |  |  |  |  |  |  |  |
| Beta-blocker | 28,309 (20) | 1,057 (12) | 6,546 (13) | 11,112 (19) | 3,736 (27) | 2,199 (31) | 641 (28) | 118 (24) | 2,900 (45) |
| RASi | 38,256 (26) | 1,383 (16) | 10,086 (21) | 15,571 (27) | 4,894 (35) | 2,681 (37) | 761 (34) | 104 (21) | 2,776 (43) |
| Statin | 47,177 (32) | 2,041 (24) | 12,408 (25) | 19,063 (33) | 5,699 (41) | 3,162 (44) | 992 (44) | 213 (44) | 3,599 (56) |
| **Smoking status** |  |  |  |  |  |  |  |  |  |
| Non-smoker | 56,379 (39) | 3,131 (36) | 14,175 (29) | 24,395 (43) | 6,523 (46) | 3,546 (49) | 1,234 (54) | 245 (50) | 3,130 (49) |
| Ex-smoker | 43,623 (30) | 2,268 (26) | 11,110 (23) | 19,118 (34) | 5,135 (37) | 2,608 (36) | 745 (33) | 171 (35) | 2,468 (39) |
| Current smoker | 45,169 (31) | 3,279 (38) | 23,792 (48) | 13,555 (24) | 2,371 (17) | 1,016 (14) | 288 (13) | 70 (14) | 798 (12) |
| **Admission year** |  |  |  |  |  |  |  |  |  |
| 2014-15 | 57,393 (40) | 4,711 (54) | 18,638 (38) | 21,785 (38) | 5,532 (39) | 2,916 (41) | 900 (40) | 190 (39) | 2,721 (43) |
| 2016-17 | 54,490 (38) | 2,356 (27) | 18,714 (38) | 21,943 (38) | 5,331 (38) | 2,729 (38) | 883 (39) | 174 (36) | 2,360 (37) |
| 2018-19 | 33,288 (23) | 1,611 (19) | 11,725 (24) | 13,340 (23) | 3,166 (23) | 1,525 (21) | 484 (21) | 122 (25) | 1,315 (21) |

* Missing eGFR and no code indicating prevalent chronic renal failure

** Anyone with a code indicating prevalent chronic renal failure, regardless of what eGFR stage the SCr recorded within 24 hours of admission corresponds to

eGFR ranges (mL/min/1.73m^2^): range 1 (≥90), range 2 (60-89), range 3a (45-59), range 3b (30-44), range 4 (15-29), and range 5 (0-14)

### **Supplementary table 10.** Description of people with coded chronic renal failure, stratified by the eGFR stage corresponding to the SCr measured within 24 hours of hospitalisation (all hospitals, N=209)

|  | **Total** | **Missing eGFR** | **Range 1** | **Range 2** | **Range 3a** | **Range 3b** | **Range 4** | **Range 5** |
| --- | --- | --- | --- | --- | --- | --- | --- | --- |
| **N= (row %)** | **18,924 (100)** | **542 (3)** | **295 (2)** | **2,114 (1)** | **3,090 (2)** | **5,116 (27)** | **4,953 (26)** | **2,814 (15)** |
| **Female** | 7,165 (38) | 167 (31) | 71 (24) | 851 (40) | 1,186 (38) | 2,044 (40) | 1,871 (38) | 975 (35) |
| **Age in years, mean (SD)** | 78 (12) | 73 (13) | 61 (11) | 75 (12) | 78 (11) | 80 (10) | 80 (11) | 73 (12) |
| **Age category in years (%)** |  |  |  |  |  |  |  |  |
| <50 | 396 (2) | 24 (4) | 40 (14) | 58 (3) | 43 (1) | 57 (1) | 74 (1) | 100 (4) |
| 50-59 | 1,192 (6) | 59 (11) | 92 (31) | 166 (8) | 140 (5) | 186 (4) | 230 (5) | 319 (11) |
| 60-69 | 2,517 (13) | 118 (22) | 99 (34) | 367 (17) | 389 (13) | 484 (9) | 516 (10) | 544 (19) |
| 70-79 | 5,295 (28) | 135 (25) | 48 (16) | 685 (32) | 907 (29) | 1,392 (27) | 1,266 (26) | 862 (31) |
| 80+ | 9,524 (50) | 206 (38) | 16 (5) | 838 (40) | 1,611 (52) | 2,997 (59) | 2,867 (58) | 989 (35) |
| **Ethnicity** |  |  |  |  |  |  |  |  |
| White | 16,276 (86) | 424 (78) | 248 (84) | 1,886 (89) | 2,751 (89) | 4,557 (89) | 4,291 (87) | 2,119 (75) |
| Black | 410 (2) | 33 (6) | 7 (2) | 27 (1) | 45 (1) | 73 (1) | 98 (2) | 127 (5) |
| Asian | 1,795 (9) | 62 (11) | 30 (10) | 156 (7) | 238 (8) | 392 (8) | 456 (9) | 461 (16) |
| Mixed/other | 443 (2) | 23 (4) | 10 (3) | 45 (2) | 56 (2) | 94 (2) | 108 (2) | 107 (4) |
| **Comorbidities** |  |  |  |  |  |  |  |  |
| Angina | 7,391 (39) | 209 (39) | 102 (35) | 800 (38) | 1,236 (40) | 2,152 (42) | 1,906 (38) | 986 (35) |
| Cerebrovascular disease | 3,235 (17) | 85 (16) | 56 (19) | 341 (16) | 542 (18) | 880 (17) | 881 (18) | 450 (16) |
| COPD | 4,278 (23) | 130 (24) | 92 (31) | 596 (28) | 742 (24) | 1,135 (22) | 1,091 (22) | 492 (17) |
| Diabetes mellitus | 9,093 (48) | 255 (47) | 117 (40) | 773 (37) | 1,289 (42) | 2,431 (48) | 2,594 (52) | 1,634 (58) |
| Heart failure | 3,962 (21) | 105 (19) | 54 (18) | 329 (16) | 559 (18) | 1,196 (23) | 1,259 (25) | 460 (16) |
| Hypercholesterolaemia | 6,953 (37) | 220 (41) | 140 (47) | 837 (40) | 1,192 (39) | 1,892 (37) | 1,701 (34) | 971 (35) |
| Hypertension | 13,773 (73) | 399 (74) | 195 (66) | 1,497 (71) | 2,276 (74) | 3,748 (73) | 3,597 (73) | 2,061 (73) |
| Previous MI | 6,881 (36) | 208 (38) | 94 (32) | 695 (33) | 1,092 (35) | 1,930 (38) | 1,866 (38) | 996 (35) |
| Peripheral vascular disease | 2,436 (13) | 80 (15) | 54 (18) | 234 (11) | 352 (11) | 653 (13) | 655 (13) | 408 (14) |
| Kidney failure | 18,924 (100) | 542 (100) | 295 (100) | 2,114 (100) | 3,090 (100) | 5,116 (100) | 4,953 (100) | 2,814 (100) |
| **Co-prescriptions** |  |  |  |  |  |  |  |  |
| Beta-blocker | 8,889 (47) | 236 (44) | 113 (38) | 841 (40) | 1,393 (45) | 2,469 (48) | 2,446 (49) | 1,391 (49) |
| RASi | 8,713 (46) | 231 (43) | 135 (46) | 1,018 (48) | 1,637 (53) | 2,626 (51) | 2,207 (45) | 859 (31) |
| Statin | 11,198 (59) | 294 (54) | 148 (50) | 1,183 (56) | 1,847 (60) | 3,021 (59) | 2,999 (61) | 1,706 (61) |
| **Smoking status** |  |  |  |  |  |  |  |  |
| Non-smoker | 9,192 (49) | 252 (46) | 94 (32) | 980 (46) | 1,474 (48) | 2,491 (49) | 2,434 (49) | 1,467 (52) |
| Ex-smoker | 7,618 (40) | 195 (36) | 101 (34) | 828 (39) | 1,293 (42) | 2,137 (42) | 2,032 (41) | 1,032 (37) |
| Current smoker | 2,114 (11) | 95 (18) | 100 (34) | 306 (14) | 323 (10) | 488 (10) | 487 (10) | 315 (11) |

eGFR ranges (mL/min/1.73m^2^): range 1 (≥90), range 2 (60-89), range 3a (45-59), range 3b (30-44), range 4 (15-29), and range 5 (0-14)

### **Supplementary table 11.** Association between eGFR stage and invasive versus conservative cardiac management in the overall study population (STEMI and NSTEMI hospitalisations), stratified by centres’ PCI availability

| **eGFR range at first hospital** | **No. (row %)** | **Total** | **Age and sex adjusted**  **OR (95% CI)** | **Adjusted***  **OR (95%CI)** | **Adjusted** predicted percent (95% CI)** |
| --- | --- | --- | --- | --- | --- |
| **PCI not available**  **(N=120 centres)** |  |  |  |  |  |
| Missing eGFR | 2,098  (64) | 3,259 | 0.49  (0.45-0.54) | 0.49  (0.45-0.54) | 57.9  (56.4-59.5) |
| 1 | 19,259  (86) | 22,451 | 0.79  (0.75-0.83) | 0.81  (0.77-0.85) | 66.7  (65.9-67.4) |
| 2 | 26,745  (70) | 38,019 | 1  (reference) | 1  (reference) | 70.2  (69.8-70.6) |
| 3a | 6,235  (53) | 11,861 | 0.71  (0.67-0.74) | 0.72  (0.69-0.76) | 64.7  (64.0-65.4) |
| 3b | 2,650  (37) | 7,177 | 0.44  (0.41-0.46) | 0.46  (0.44-0.49) | 56.9  (55.9-57.8) |
| 4 | 495  (19) | 2,544 | 0.17  (0.16-0.19) | 0.19  (0.17-0.21) | 40.7  (38.7-42.6) |
| 5 | 87  (20) | 432 | 0.11  (0.08-0.14) | 0.12  (0.09-0.15) | 32.3  (28.0-36.7) |
| Chronic renal failure | 3,481  (42) | 8,379 | 0.40  (0.38-0.43) | 0.45  (0.42-0.47) | 56.1  (55.2-57.1) |
| **PCI available sometimes**  **(N=38 centres)** |  |  |  |  |  |
| Missing eGFR | 1,018  (76) | 1,342 | 0.48  (0.42-0.56) | 0.47  (0.41-0.55) | 68.0  (65.6-70.3) |
| 1 | 13,340  (92) | 14,523 | 0.80  (0.74-0.87) | 0.79  (0.73-0.86) | 75.7  (74.7-76.8) |
| 2 | 16,913  (79) | 21,398 | 1  (reference) | 1  (reference) | 78.9  (78.4-79.4) |
| 3a | 4,157  (62) | 6,725 | 0.65  (0.61-0.69) | 0.67  (0.63-0.72) | 73.4  (72.6-74.2) |
| 3b | 1,974  (47) | 4,196 | 0.42  (0.39-0.45) | 0.45  (0.42-0.49) | 67.3  (66.1-68.4) |
| 4 | 454  (32) | 1,437 | 0.22  (0.19-0.25) | 0.24  (0.21-0.27) | 56.3  (54.1-58.5) |
| 5 | 68  (28) | 242 | 0.12  (0.09-0.17) | 0.13  (0.09-0.18) | 45.5  (39.9-51.1) |
| Chronic renal failure | 1,942  (44) | 4,388 | 0.27  (0.25-0.29) | 0.33  (0.30-0.36) | 61.9  (60.7-63.1) |
| **PCI always available**  **(N=51 centres)** |  |  |  |  |  |
| Missing eGFR | 7,864  (93) | 8,412 | 0.82  (0.75-0.91) | 0.83  (0.75-0.92) | 91.4  (90.8-92.0) |
| 1 | 47,493  (97) | 48,851 | 0.96  (0.89-1.03) | 0.92  (0.86-0.99) | 92.1  (91.7-92.5) |
| 2 | 52,321  (92) | 56,873 | 1  (reference) | 1  (reference) | 92.6  (92.4-92.8) |
| 3a | 11,655  (83) | 13,997 | 0.67  (0.63-0.70) | 0.71  (0.67-0.75) | 90.2  (89.9-90.6) |
| 3b | 5,292  (74) | 7,158 | 0.45  (0.42-0.48) | 0.50  (0.47-0.53) | 87.3  (86.7-87.8) |
| 4 | 1,405  (62) | 2,266 | 0.27  (0.25-0.30) | 0.31  (0.28-0.34) | 82.1  (81.0-83.3) |
| 5 | 309  (64) | 485 | 0.17  (0.14-0.21) | 0.19  (0.16-0.24) | 76.0  (73.0-79.0) |
| Chronic renal failure | 4,462  (70) | 6,376 | 0.28  (0.26-0.30) | 0.37  (0.35-0.40) | 84.4  (83.7-85.0) |

* Adjusted for age (continuous), sex, ethnicity (White, Black, Asian, Mixed/other), comorbidities (previous MI, angina, hypertension, hypercholesterolaemia, peripheral vascular disease, cerebrovascular disease, COPD, heart failure, type 2 diabetes mellitus), prevalent prescriptions (RASi, beta blockers, statins), smoking status, and admission year (2014-15, 2016-17, 2018-19)

** Adjusted predicted percentages derived using the adjusted model

eGFR ranges (mL/min/1.73m^2^): range 1 (≥90), range 2 (60-89), range 3a (45-59), range 3b (30-44), range 4 (15-29), and range 5 (0-14)

# **SUPPLEMENTARY FIGURES**

### **Supplementary figure 1.** Variation in AMI treatment (angiography and/or PCI) at the centre-level (N=209 centres total), for (a) STEMI and (b) NSTEMI.


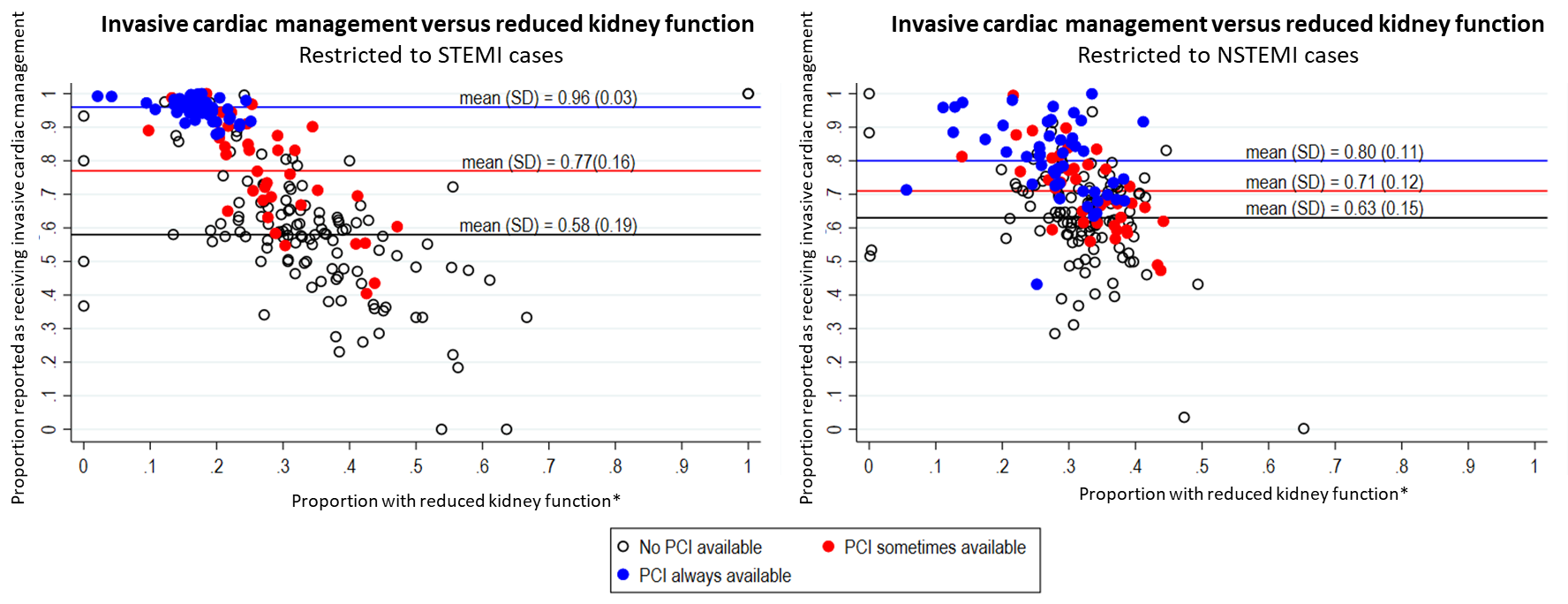


*Reduced kidney function defined as eGFR ranges 3a-5 or coded renal impairment

### **Supplementary figure 2.** Centre-level variation in the proportion receiving invasive cardiac treatment versus the proportion with clinically important characteristics (aged ≥80 years, previous myocardial infarction, prevalent diabetes mellitus, and prevalent heart failure)


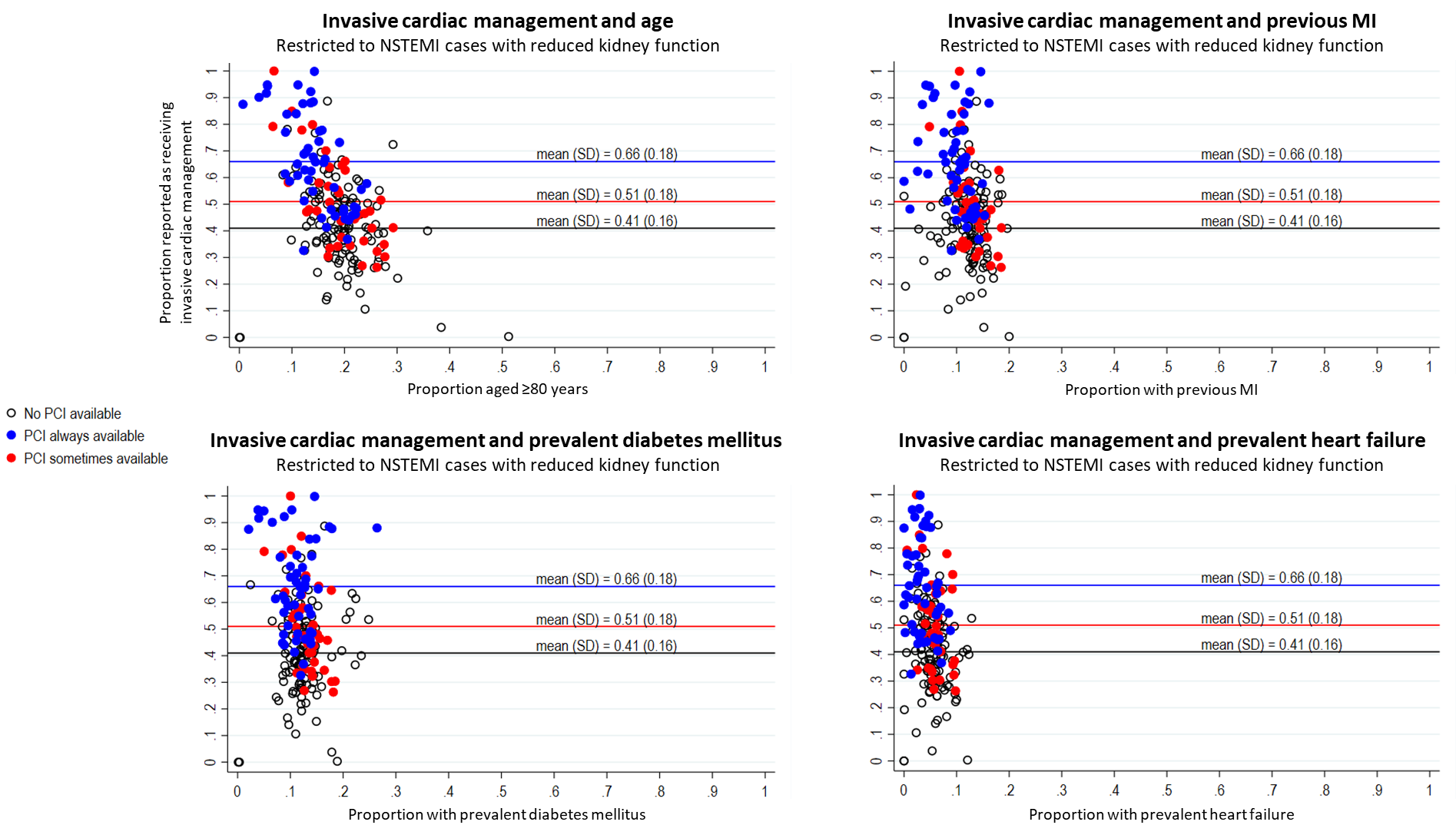

Supplement: online supplemental file 1 [file bmjopen-15-5-s001.docx]
