# Supplementary material for: Variation in acute myocardial infarction management by kidney function across hospitals in England: a cross-sectional study using the Myocardial Ischaemia National Audit Project (MINAP)
Source: BMJ Open. 2025 May 16;15(5):e096991. doi: 10.1136/bmjopen-2024-096991 (PMC12086913; doi:10.1136/bmjopen-2024-096991)
Supplement: Supplementary file 2 [file bmjopen-2024-096991.reviewer_comments.pdf]

PEER REVIEW HISTORY

BMJ Open publishes all reviews undertaken for accepted manuscripts. Reviewers are asked to complete a checklist review form and are provided with free text boxes to elaborate on their assessment. These free text comments are reproduced below.

ARTICLE DETAILS

Title (Provisional)

Variation in acute myocardial infarction management by kidney function across hospitals in England: a cross-sectional study using the Myocardial Ischaemia National Audit Project (MINAP)

Authors

Bidulka, Patrick; Weston, Clive; de Belder, Mark; Deanfield, John; Konstant-Hambling, Rob; Grieve, Richard; Adlam, David; Nitsch, Dorothea

---

VERSION 1 - REVIEW

**Reviewer**	**1**
**Name**	**Kampmann, Jan**
**Affiliation**	**Hospital of Southern Jutland Sonderborg Branch, Internal medicine**
**Date**	**30-Dec-2024**
**COI**	**None**

Thank you for giving me the opportunity to review your manuscript "Variation in acute myocardial infarction management by

kidney function across cardiology centres in England: a

cross-sectional study using the Myocardial Ischaemia

National Audit Project (MINAP)

This manuscript describes the variation of treatment of AMI in patients with renal impairment across England.

I find the study very important and well designed.

I have only minor comments:

I think it is problematic to use stage 1 and 2 as eGFR stage. The classical definition of CKD stage 1-2 requires som degree of renal impairment such as elevated ACR. I know that you go by the eGFR measured in secondary care, however a footnote to clearify that eGFR stage 1-2 are not per definition renal impairment would clarify this matter.

I suggest to mention that diagnosing AMI in patients with renal impairment is difficult due to atypical symptoms, elevated troponin at baseline and atypical ECG changes. This may be seen as a limitation. Is there any data on the validity of AMI diagnosis in patients with renal impairment in England?

**Reviewer**	**2**
**Name**	**Wakabayashi, Kohei**
**Affiliation**	**Showa University Koto Toyosu Hospital**
**Date**	**05-Jan-2025**
**COI**	**None**

Patrick et al. investigated the variation in AMI treatment by kidney impairment across the hospitals in England. The primary endpoint was invasive cardiac management defined as angiography and, if indicated, primary PCI or CABG. They assessed the variation at the hospital-level and the individual-level using the data from the Myocardial Ischemia National Audit Project (MINAP). As their hypothesis, they found substantial variation of AMI treatment, particularly among the patients hospitalized for NSTEMI with impaired kidney function. The topic is interesting and important for perspectives. The manuscript is well written with standard manner. There are some comments.

“Introduction” is relatively long and difficult to understand the aim of this study for general cardiologists. It should be better to shorten “Introduction” and make the main purpose clear.

The current guidelines recommend to apply the same diagnostic and therapeutic strategies in patients with chronic kidney disease (CKD) as for patients with normal kidney function, but evidence from RCTs is lacking [European Heart Journal (2023) 44, 3720–3826]. As the authors mentioned, it may be the reason of variation of AMI treatment in NSTEMI with CKD. It is known that patients with acute coronary syndrome (ACS) and CKD receive less interventional treatment and have a worse prognosis than patients without CKD. Furthermore, data from observational and registry-based studies indicate that ACS patients with moderate to severe CKD have a better prognosis with early revascularization than with medical therapy alone. To increase the value of this manuscript, variation of mortality across 51 hospitals should be shown according to invasive or conservative cardiac management. The results should be informative and give benefit for perspectives.

**Reviewer**	**3**
**Name**	**Dack, Kyle**
**Affiliation**	**University of Bristol, MRC Integrative Epidemiology Unit**
**Date**	**28-Jan-2025**

COI **None**

The methodology and statistical analysis are described clearly and the approach is valid. Below are mostly suggestions, but there are three major points of concern:

1. I think the authors may have selected covariables to adjust for confounding, but this is not explained or discussed, in fact confounding is barely mentioned at all. The risk of confounding is a primary limitation for most observational studies due to unmeasured or residual confounding, and the authors are in the best position to judge this – I suggest explaining the approach in the methods and adding as discussion in the strengths or limitations.

2. Approximately 20% of patients were excluded from the individual level analyses due to missing data. Which data was missing and the structure of missingness should be explored in more detail because it impacts the validity of a complete case analysis. I suggest adding a supplement which reports (a) the % missing of each variable and (b) compares descriptives (such as in table 1) between complete cases and incomplete cases to determine if missingness is correlated with any variables.

3. The study is described as cross-sectional, but the exposure is recorded at time of admission, and the outcome occurs later. This seems to be a retrospective cohort design?

Abstract

1. The acronyms AMI and NSTEMI are not defined in the first instance they are used in the abstract.

Methods

2. Study population: Given the 5 year time frame, is it possible that patients were admitted on multiple occasions for AMI? Either to the same hospital, or a different hospital? If so, were there any exclusions or checks for this, or did the analysis allow for multiple admittances? I don't think it makes a huge difference, but for transparency I think the methods should discuss this possibility and explain the approach the authors took.

3. "We categorised people with missing eGFR separately, but assumed they did not have kidney impairment in analyses which stratified by kidney function" Could the authors explain a little more the justification behind this? The previous paragraph stated that individuals with missing covariates were excluded, why was this not also applied to those with missing exposure data – was there another way of inferring kidney impairment?

4. A number of covariates are defined – but why were these selected and adjusted for? If they are considered confounders which could affect both kidney function and treatment choice, I suggest explicitly stating this.

5. Something missing from either the exposure or covariate section is an explanation of cardiology centers. This is a key variable in the mixed effect models, but it is not explained

how the variable is formed – a category for each center, or was there some form of hierarchy?

6. The modelling strategy is difficult to follow due to repetition, differing numberings, and the length of the sentence. I suggest making this easier to read by splitting the sentences and either starting each with something simple such as “Model 1: ..” or by removing the repetition, for example “Model 2 was the same as model 1, with the addition of all covariates as fixed effects.”

7. “since the interpretation of odds ratios with a common outcome is difficult” Suggest rephrasing to “because this will make the effect estimate easier to interpret” or similar.

8. In total, the statistical analysis section describes 5 or 6 different methods which were applied to achieve different goals, such as stratifying by eGFR and covariates. Readers may find this hard to follow or surprising, because the study aim quite non-specific “describe treatment variation in England among those treated for AMI with kidney impairment. Specifically, we aimed to describe this variation at the hospital (centre)-level and the individual-level”. The paper would be clearer if a full list of study objectives was briefly stated either after the study aims, in the study design, or the very beginning of the results.

Results

9. Table 2. Suggest removing the % from “total” because it must be 100%. The APP 95% CI will be more readable as a single column.

10. “After adjusting for centre as a random effect and covariables as fixed effects” it is not clear to me what the purpose of the covariables is – whether its to minimize the risk of confounding, or whether these covariables themselves are of interest.

11. “The intracluster correlation coefficients from our mixed effect models 1, 2, and 3…” please add that the reported results are from model 3.

VERSION 1 - AUTHOR RESPONSE

**Peer Review Comment**	**Authors’ response**	**Edit location in revised manuscript**
**Reviewer 1 – Mr Jan Kampmann**		
Thank you for giving me the opportunity to review your manuscript "Variation in acute myocardial infarction management by kidney function across	Thank you for your interest in our manuscript.	N/A

cardiology centres in England: a cross-sectional study using the Myocardial Ischaemia National Audit Project (MINAP)<br><br>This manuscript describes the variation of treatment of AMI in patients with renal impairment across England.<br><br>I find the study very important and well designed.<br><br>I have only minor comments:		
I think it is problematic to use stage 1 and 2 as eGFR stage. The classical definition of CKD stage 1-2 requires som degree of renal impairment such as elevated ACR. I know that you go by the eGFR measured in secondary care, however a footnote to clearify that eGFR stage 1-2 are not per definition renal impairment would clarify this matter.	We agree with the reviewer that an eGFR between 90-120 and between 60-89mL/min/1.73m^2 does not qualify as CKD stages 1 and 2, respectively.<br><br>We were careful throughout the manuscript to describe these categorisations as eGFR stages, rather than CKD stages. However, we have edited the text throughout to read "eGFR ranges" to emphasise that we are not describing CKD stages.<br><br>We have also replaced the term "kidney impairment" with "reduced kidney function". This emphasises that we are merely observing decreased kidney function as measured by the eGFR.<br><br>We also highlighted in the methods (exposure of interest) that we did not have other measures of kidney function like ACR, which mean we cannot define CKD status. Instead, we are defining eGFR ranges.	Methods (exposure), page 8.<br><br>Edits throughout manuscript changing 'kidney impairment' to 'reduced kidney function'.

I suggest to mention that diagnosing AMI in patients with renal impairment is difficult due to atypical symptoms, elevated troponin at baseline and atypical ECG changes. This may be seen as a limitation. Is there any data on the validity of AMI diagnosis in patients with renal impairment in England?	Thank you for raising this important point. We have conducted previous work using the same data sources from England showing that AMI case ascertainment is incomplete across different secondary care datasets, and this is impacted by kidney disease status as defined in primary care (a better estimate of someone's baseline kidney function).[1]<br>We have cited this work in the manuscript already. However, we have added text in the discussion to be more explicit in our reference to this potential limitation.<br>The new text now reads:<br>"These data were unlinked to other routinely collected health datasets. We know from previous work that AMI case ascertainment is associated with baseline kidney function,[1] and is incomplete with MINAP alone,[1-3] particularly since MINAP focuses on capturing type 1 AMI.[4]"	Discussion (Limitations), page 19.
**Reviewer 2 – Dr Kohei Wakabayashi**		
Patrick et al. investigated the variation in AMI treatment by kidney impairment across the hospitals in England. The primary endpoint was invasive cardiac management defined as angiography and, if indicated, primary PCI or CABG. They assessed the variation at the hospital-level and the individual-level using the data from the Myocardial Ischemia National Audit Project	Thank you for your interest in our manuscript.	N/A

(MINAP). As their hypothesis, they found substantial variation of AMI treatment, particularly among the patients hospitalized for NSTEMI with impaired kidney function. The topic is interesting and important for perspectives. The manuscript is well written with standard manner. There are some comments.		
"Introduction" is relatively long and difficult to understand the aim of this study for general cardiologists. It should be better to shorten "Introduction" and make the main purpose clear.	Thank you for this suggestion. We have edited the text to shorten the introduction. We have also added specific objectives following the main aim of the paper at the end of the introduction to make the main purpose more clear, as suggested by yourself and the third reviewer.	Introduction, pages 6-7.
The current guidelines recommend to apply the same diagnostic and therapeutic strategies in patients with chronic kidney disease (CKD) as for patients with normal kidney function, but evidence from RCTs is lacking [European Heart Journal (2023) 44, 3720–3826]. As the authors mentioned, it may be the reason of variation of AMI treatment in NSTEMI with CKD. It is known that patients with acute coronary syndrome (ACS) and CKD receive less interventional treatment and have a worse prognosis than patients without CKD. Furthermore,	We appreciate that understanding the comparative effectiveness of early invasive versus conservative management strategies is of high clinical importance.<br><br>We did not report variation in mortality across centres, as we are using only a single, unlinked cardiac audit which has been shown to be incomplete with respect to AMI hospitalisations (STEMI and NSTEMI) compared with data from multiple linked data sources.[1,2]<br><br>We have edited the text in the Discussion (Future research) to highlight the importance of investigating the relative effectiveness of these managements strategies on important outcomes like mortality. The edited text reads:	Discussion (future research), page 20.

data from observational and registry-based studies indicate that ACS patients with moderate to severe CKD have a better prognosis with early revascularization than with medical therapy alone. To increase the value of this manuscript, variation of mortality across 51 hospitals should be shown according to invasive or conservative cardiac management. The results should be informative and give benefit for perspectives.	"The treatment variation we described in this study should be further explored in linked secondary care data to understand if this variation can be exploited in a natural experiment, comparing invasive versus conservative NSTEMI management strategies in people with reduced kidney function and/or CKD for important outcomes like mortality."<br><br>In previous work, we did report associations between early invasive versus conservative NSTEMI management using these linked data sources. However, we did not draw any causal conclusions, since the study was underpowered and likely subject to substantial residual confounding bias.[5]<br><br>In on-going work which builds on the analyses in this manuscript, we are using the hospital-level variation in early invasive versus conservative NSTEMI management strategies to compare these treatments for several important outcomes, including death, using advanced methods which will reduce the risk of bias impacting our results.	
**Reviewer 3 – Dr Kyle Dack**		
The methodology and statistical analysis are described clearly and the approach is valid. Below are mostly suggestions, but there are three major points of concern:	Thank you for taking the time to review our manuscript.	N/A
1. I think the authors may have selected covariables to adjust for confounding, but this is not explained or	We agree that confounding is a major limitation in any observational analysis. We have added a sentence to explain in the	Methods (Covariables), page 9.

discussed, in fact confounding is barely mentioned at all. The risk of confounding is a primary limitation for most observational studies due to unmeasured or residual confounding, and the authors are in the best position to judge this – I suggest explaining the approach in the methods and adding as discussion in the strengths or limitations.	Covariables section that we expect these variables to act as confounders when looking at the relationship between reduced kidney function and AMI management strategy.<br><br>We also added a description in the limitations section of the discussion to acknowledge the impact of residual confounding, particularly by frailty, in our results. This paragraph reads:<br><br>"We adjusted for measured confounders in our multivariable models quantifying the association between reduced kidney function and AMI management strategy. However, our findings are likely impacted by residual confounding by factors such as frailty, which influence both degree of kidney impairment and AMI management strategies people receive in hospital."	Methods (Analysis), page 12.<br><br>Discussion (Limitations), page 20.
2. Approximately 20% of patients were excluded from the individual level analyses due to missing data. Which data was missing and the structure of missingness should be explored in more detail because it impacts the validity of a complete case analysis. I suggest adding a supplement which reports (a) the % missing of each variable and (b) compares descriptives (such as in table 1) between complete cases and incomplete cases to determine if missingness is correlated with any variables.	We appreciate the concern about high levels of missing covariable data in our analysis. We agree with the reviewer that this limitation needs to be better described and acknowledged as a limitation in the discussion.<br><br>We have added two descriptive tables to the supplement:<br><br>Supplementary table 5: Describes the overall population (N=361,259) hospitalised for NSTEMI, and stratifies the baseline table, including all covariables, by complete and incomplete case.<br><br>Supplementary table 6: The same as Supp table 5 except the population is restricted to people hospitalised at hospitals with PCI always available (N=180,967). This	Discussion (limitations), page 20.<br><br>Supplementary tables 5 and 6.

	was the population of focus for the main analyses in objective 3 (quantifying the association between kidney function and AMI management strategy).<br><br>In these tables, we see that missing ethnicity and smoking status are the variables with the most missing data (14% and 6% missing) leading to approximately 20% of the sample being excluded.<br><br>People with missing covariable data tended to have slightly lower prevalences of recorded comorbidities. Thus, we have added as a limitation in the discussion section that by doing a complete case analysis, we may have overestimated any disparities in the provision of invasive versus conservative AMI management, since receiving invasive versus conservative AMI management is associated with better health status. The new text in the discussion reads:<br><br>"For all other missing covariable data, we used a complete case analysis approach. People with missing covariable data tended to have lower recorded prevalences of comorbidities. By excluding these people, we may have overestimated variation in AMI management, since healthier people tend to receive invasive versus conservative cardiac management."	
3. The study is described as cross-sectional, but the exposure is recorded at time of admission, and the outcome occurs later. This seems to be a retrospective cohort design?	We agree that there may be time between the serum creatinine measure and the management strategy being implemented in this study population.<br><br>Unfortunately, these audit data do not have complete information regarding the exact timing of the	Methods (study design and data source), page 7).<br><br>Discussion (Limitations), page 20).

	invasive cardiac management (angiography, PCI, or CABG). So we are unable to determine if the SCr measure and the invasive management occur on the same day or whether there is a gap between the exposure and outcome.<br><br>We have edited the study design section in the methods to explain that we consider it a cross-sectional study, looking at the entire hospitalisation as a snapshot of care. The end of the study design and data source section now reads:<br><br>"We considered the entire AMI hospitalisation as a snapshot of care received for each person included in this cross-sectional study."<br><br>We have also added in the limitation section that we were unable to incorporate the potential time between the exposure and outcome to analyse this study as a cohort study. This paragraph reads:<br><br>"Finally, we did not incorporate the potential time between the exposure measurement (serum creatinine laboratory test) and AMI management in the study design and analysis, since the timings of the exposure and outcomes were incompletely described in these data."	
Abstract<br>1. The acronyms AMI and NSTEMI are not defined in the first instance they are used in the abstract.	We have defined these in the abstract, and made sure all other acronyms are properly defined.	Abstract, page 3

Methods<br>2. Study population: Given the 5 year time frame, is it possible that patients were admitted on multiple occasions for AMI? Either to the same hospital, or a different hospital? If so, were there any exclusions or checks for this, or did the analysis allow for multiple admittances? I don't think it makes a huge difference, but for transparency I think the methods should discuss this possibility and explain the approach the authors took.	We included only the first hospitalisation in the study period for each person in the study. We expect subsequent hospitalisations to be managed differently to a first hospitalisation.<br>However, we acknowledge that some people had previously experienced AMI – this was included as a potential confounder in the multivariable model.<br>We have edited the methods section (study population) to make this more clear. The study population flow diagram also showed that we excluded 39,447 re-hospitalisations for AMI following the first AMI hospitalisation. We edited the study population diagram to make it clear that in the first box, it is 450,264 AMI hospitalisations, not people, which we started with. After excluding re-hospitalisations, unstable angina hospitalisations, and other diagnoses, we were left with 361,259 AMI hospitalisations in unique individuals.	Methods (study population), page 8.<br>Figure 1.
3. "We categorised people with missing eGFR separately, but assumed they did not have kidney impairment in analyses which stratified by kidney function" Could the authors explain a little more the justification behind this? The previous paragraph stated that individuals with missing covariates were excluded, why was this not also applied to those with missing exposure data – was there another way of	We appreciate the reviewer's concern. In previous work, we showed that we could accurately estimate prevalence of decreased kidney function (eGFR<60mL/min/1.73m^2) and renal replacement therapy in primary care electronic health record data compared with survey and registry data (gold-standard).[6] These analyses assumed that missing eGFR represented no reduced kidney function, since the missingness was associated with better health status.<br>We acknowledge that we are using secondary care audit data, and not	Discussion (limitations), page 19.<br>Supplementary tables 7 and 8.

inferring kidney impairment?	primary care EHR data. Thus, the findings from our previous study may not hold in secondary care audit data.<br><br>We have added two tables which describe differences in baseline characteristics between those with missing and non-missing serum creatinine test results in the MINAP data, for the overall population and the population hospitalised where PCI is always available (supplementary tables 7-8).<br><br>We found that people with missing serum creatinine (eGFR) were on average younger and with a lower proportion of all measured comorbidities. Thus, rather than excluding this large proportion of people with missing kidney function, we made the assumption these people did not have reduced kidney function, as we regularly do when using primary care data.<br><br>Reassuringly, in our analyses of the association between eGFR stage and AMI management strategy, we found that those with missing eGFR (kept as a separate exposure group) had similar results to those people who we would not categorise as having reduced kidney function (eGFR ranges 3a-5 and coded renal failure).<br><br>We acknowledge that this assumption may lead to some misclassification of the exposure. We have added a brief discussion of this in the limitation section of the discussion.	
4. A number of covariates are defined – but why were these selected and adjusted for? If they are considered confounders	We have revised the text to clearly explain that the covariables are thought to be potential confounders of the association of interest, and thus were included as independent	Methods (covariables), page 9.

which could affect both kidney function and treatment choice, I suggest explicitly stating this.	variables in the multivariable model.	Methods (analysis), page 11.<br><br>Discussion (limitations), page 20.
5. Something missing from either the exposure or covariate section is an explanation of cardiology centers. This is a key variable in the mixed effect models, but it is not explained how the variable is formed – a category for each center, or was there some form of hierarchy?	We used the terms hospitals and cardiology centres interchangeably. However, the reviewer has rightly highlighted that this will lead to confusion.<br><br>We have edited the text throughout to refer only to hospitals, rather than cardiology centres.<br><br>We edited the Covariates section to explain that MINAP data distinguishes between different hospitals, rather than grouping hospitals at the NHS Trust-level. The individual hospitals and how we categorised them based on PCI service availability are provided in Supplementary table 3.	Methods (covariables), page 9-10.<br><br>'Cardiology centres' changed to 'hospitals' throughout.
6. The modelling strategy is difficult to follow due to repetition, differing numberings, and the length of the sentence. I suggest making this easier to read by splitting the sentences and either starting each with something simple such as "Model 1: .." or by removing the repetition, for example "Model 2 was the same as model 1, with the addition of all covariates as fixed effects."	Thank you for the suggestion – we have edited the text to make this description clearer using the second option the reviewer suggests.	Methods (Analysis), page 11-12.
7. "since the interpretation of odds ratios with a common outcome is difficult" Suggest	Thank you for the suggestion. We have edited the text as you suggest. The text now reads:	Methods (Analysis), page 12.

rephrasing to "because this will make the effect estimate easier to interpret" or similar.	"We calculated the adjusted predicted percentages of receiving invasive versus conservative cardiac management by eGFR range from model 3 to understand individual-level variation in AMI treatment, since percentages are easier to interpret than odds ratios.[7,8]"	
8. In total, the statistical analysis section describes 5 or 6 different methods which were applied to achieve different goals, such as stratifying by eGFR and covariates. Readers may find this hard to follow or surprising, because the study aim quite non-specific "describe treatment variation in England among those treated for AMI with kidney impairment. Specifically, we aimed to describe this variation at the hospital (centre)-level and the individual-level". The paper would be clearer if a full list of study objectives was briefly stated either after the study aims, in the study design, or the very beginning of the results.	We agree that outlining specific objectives in the introduction and structuring the analysis and results sections by objectives makes the paper easier to read.<br><br>We have added a list of specific objectives just after the overall aim statement, which are the following:<br><br>1. Describe the population hospitalised for AMI and the crude proportion managed with invasive versus conservative cardiac management (stratified by AMI subtype and kidney function) aggregated at the hospital-level.<br><br>2. Repeat the objective 1 descriptive analysis at the individual-level.<br><br>3. Describe the association between reduced kidney function and AMI management strategy for STEMI and NSTEMI cases separately.	Introduction, page 7.<br><br>Methods (analysis), pages 10-12.<br><br>Results, pages 12-16.<br><br>Supplementary tables (re-ordered according to how they are now presented in the main text).
Results<br>9. Table 2. Suggest removing the % from "total" because it must be 100%. The APP 95% CI will be more readable as a single column.	We have removed the % from the total column, and re-titled the column to be "Row total" to indicate to readers that this is the row total for each eGFR category.<br><br>Further, we edited the table to be one column for all point estimates and 95% CI.	Table 2.

10. “After adjusting for centre as a random effect and covariables as fixed effects” it is not clear to me what the purpose of the covariables is – whether its to minimize the risk of confounding, or whether these covariables themselves are of interest.	As we explained in previous comments, we are adjusting for these covariables as potential confounders. We agree with the reviewer it is important to be upfront about this, and have made this clear in the methods and discussion. We are not interested in the coefficients for each covariable in the model.<br><br>We also acknowledge in the limitations section of the discussion the possibility of residual confounding.	Methods (covariables), page 9.<br><br>Methods (analysis), page 11-12.<br><br>Discussion (limitations), page 20.
11. “The intracluster correlation coefficients from our mixed effect models 1, 2, and 3…” please add that the reported results are from model 3.	We have made this edit.	Results, page 16.

References

1. Bidulka P, Scott J, Taylor DM, et al. Impact of chronic kidney disease on case ascertainment for hospitalised acute myocardial infarction: an English cohort study. *BMJ Open*. 2022;12(3):e057909. doi:10.1136/bmjopen-2021-057909
2. Herrett E, Shah AD, Boggon R, et al. Completeness and diagnostic validity of recording acute myocardial infarction events in primary care, hospital care, disease registry, and national mortality records: cohort study. *BMJ : British Medical Journal*. 2013;346:f2350. doi:10.1136/bmj.f2350
3. Coles B, Teece L, Weston C, et al. Case-ascertainment of acute myocardial infarction hospitalisations in cancer patients: a cohort study using English linked electronic health data. *European Heart Journal - Quality of Care and Clinical Outcomes*. 2021;doi:10.1093/ehjqcco/qcab045
4. Herrett E, Smeeth L, Walker L, Weston C, on behalf of the MAG. The Myocardial Ischaemia National Audit Project (MINAP). *Heart (British Cardiac Society)*. 2010;96(16):1264. doi:10.1136/hrt.2009.192328
5. Scott J, Bidulka P, Taylor DM, et al. Management and outcomes of myocardial infarction in people with impaired kidney function in England. *BMC Nephrology*. 2023/11/02 2023;24(1):325. doi:10.1186/s12882-023-03377-x
6. Iwagami M, Tomlinson LA, Mansfield KE, et al. Validity of estimated prevalence of decreased kidney function and renal replacement therapy from primary care electronic health records compared with national survey and registry data in the United Kingdom. *Nephrol Dial Transplant*. Apr 1 2017;32(suppl_2):ii142-ii150. doi:10.1093/ndt/gfw318

7. Norton EC, Dowd BE, Maciejewski ML. Odds Ratios—Current Best Practice and Use. *Jama*. 2018;320(1):84-85. doi:10.1001/jama.2018.6971
8. Bidulka P, Mathur R, Lugo-Palacios DG, et al. Ethnic and socioeconomic disparities in initiation of second-line antidiabetic treatment for people with type 2 diabetes in England: A cross-sectional study. *Diabetes, Obesity and Metabolism*. 2023;25(1):282-292. doi:https://doi.org/10.1111/dom.14874

VERSION 2 - REVIEW

**Reviewer**	**1**
**Name**	**Kampmann, Jan**
**Affiliation**	**Hospital of Southern Jutland Sonderborg Branch, Internal medicine**
**Date**	**27-Mar-2025**
**COI**	

Design and Setting section in the abstract is rather short (one sentence), however it is concise and therefore still acceptable.

**Reviewer**	**2**
**Name**	**Wakabayashi, Kohei**
**Affiliation**	**Showa University Koto Toyosu Hospital**
**Date**	**03-Apr-2025**
**COI**	

Current manuscript is acceptable for publication
